# Library screening identifies commercial drugs as potential structure correctors of abnormal apolipoprotein A-I

**DOI:** 10.1016/j.jlr.2024.100543

**Published:** 2024-04-17

**Authors:** Christina Gkolfinopoulou, Angeliki Bourtsala, Daphne Georgiadou, Anastasia-Georgia Dedemadi, Efstratios Stratikos, Angeliki Chroni

**Affiliations:** 1 Institute of Biosciences and Applications, National Center for Scientific Research “Demokritos”, Athens, Greece; 2 Department of Chemistry, National and Kapodistrian University of Athens, Athens, Greece

**Keywords:** apolipoprotein, drug screening, small molecule, protein conformation, cholesterol, atherosclerosis

## Abstract

AapoA-I, the main protein of high-density lipoprotein, plays a key role in the biogenesis and atheroprotective properties of high-density lipoprotein. We showed previously that a naturally occurring apoA-I mutation, L178P, induces major defects in protein’s structural integrity and functions that may underlie the increased cardiovascular risk observed in carriers of the mutation. Here, a library of marketed drugs (956 compounds) was screened against apoA-I[L178P] to identify molecules that can stabilize the normal conformation of apoA-I. Screening was performed by the thermal shift assay in the presence of fluorescent dye SYPRO Orange. As an orthogonal assay, we monitored the change in fluorescence intensity of 8-anilinonaphthalene-1-sulfonic acid upon binding on hydrophobic sites on apoA-I. Screening identified four potential structure correctors. Subsequent analysis of the concentration-dependent effect of these compounds on secondary structure and thermodynamic stability of WT apoA-I and apoA-I[L178P] (assessed by thermal shift assay and circular dichroism spectroscopy), as well as on macrophage viability, narrowed the potential structure correctors to two, the drugs atorvastatin and bexarotene. Functional analysis showed that these two compounds can restore the defective capacity of apoA-I[L178P] to promote cholesterol removal from macrophages, an important step for atheroprotection. Computational docking suggested that both drugs target a positively charged cavity in apoA-I, formed between helix 1/2 and helix 5, and make extensive interactions that could underlie thermodynamic stabilization. Overall, our findings indicate that small molecules can correct defective apoA-I structure and function and may lead to novel therapeutic approaches for apoA-I–related dyslipidemias and increased cardiovascular risk.

## Graphical abstract

**Figure:**
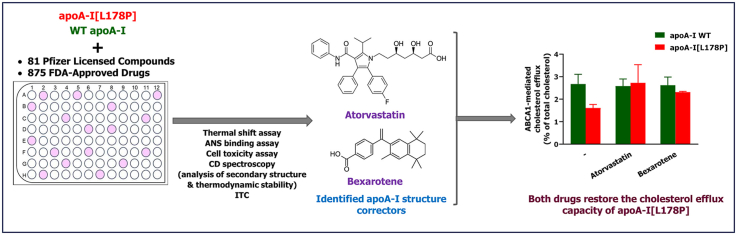

ApoA-I, the main protein component of HDL, plays a crucial role in the biogenesis and structure of HDL, contributing to its atheroprotective properties (1, 2, 3). Human apoA-I consists of 243 amino acid residues, with its main structural motif being the amphipathic α-helix (4). Studies employing X-ray crystallography, mutation analyses, and biophysical and functional assessments suggest that the N-terminal domain (residues 1–184) of lipid-free apoA-I forms a helical bundle that is crucial for protein stability (5, 6). The C-terminal domain (residues 185–243) is considered to lack a well-defined structure but plays a pivotal role in initiating lipid-binding (5, 6). A combination of distance constraints from cross-linking experiments, small-angle X-ray scattering, hydrogen-deuterium exchange, and crystallography data have yielded a consensus model for the structure of lipid-free full-length apoA-I (4). This model proposes a two-domain structure for apoA-I, with the N-terminal domain forming a helical bundle and the C-terminal domain adopting a predominantly random-coil conformation (4).

HDL biogenesis is initiated following the interaction of lipid-free apoA-I with the ABCA1 lipid transporter, resulting in the transfer of cellular phospholipids and cholesterol to apoA-I (7). As apoA-I accumulates more lipids, discoidal particles, rich in unesterified cholesterol, are formed. Subsequently, lipoprotein-associated apoA-I activates the enzyme lecithin:cholesterol acyltransferase, which esterifies cholesterol molecules, leading to the conversion of discoidal particles into mature spherical HDL particles (7). After synthesis, HDL undergoes remodeling through interactions with various cell receptors, transporters, plasma enzymes, and lipid transfer proteins (7). The interplay between apoA-I and various proteins in the HDL metabolism pathway controls plasma HDL levels and influences the atheroprotective properties of HDL, which include cholesterol efflux capacity, antioxidant, anti-inflammatory, and immune-regulating activities (1, 8).

The most extensively studied function of apoA-I is related to atheroprotection and involves the capacity of apoA-I to promote cholesterol efflux from macrophages in the artery wall (9). This process involves the interaction of lipid-free or lipid-poor apoA-I with ABCA1 (10) and the transfer of cholesterol, along with phospholipids, from macrophages to apoA-I marks the initial step of the reverse cholesterol transport pathway (11). In this pathway, cholesterol originating from peripheral cells, including macrophages, is conveyed to the liver for subsequent elimination as a component of bile. In addition to lipid-free apoA-I that promotes ABCA1-dependent cholesterol efflux, HDL-associated apoA-I can also promote cholesterol efflux mediated by the cholesterol transporter ABCG1 (12) and by the HDL receptor scavenger receptor class B type I  (13). The capacity of apoA-I to promote cholesterol efflux from macrophages has also been proposed to participate in the anti-inflammatory effects of apoA-I in these cells (14). Furthermore, apoA-I and HDL-apoA-I have been shown to exert atheroprotective and anti-inflammatory functions in endothelial cells (15, 16). Further evidence supporting the benefits of apoA-I against atherosclerosis has been provided by in vivo studies. Atherosclerosis-reducing effects were observed in mice with transgenic or adenoviral-mediated overexpression of the human *APOA1* gene, as well as in mice following administration of purified apoA-I, and it was proposed that apoA-I hinders atherosclerosis by lowering lipid and immune cell accumulation within atherosclerotic lesions (17, 18, 19). In addition to reducing atherosclerosis, apoA-I has been proposed to be protective against nonischemic cardiomyopathies, thrombosis, diabetes, as well as diseases nonrelated to the cardiovascular system, such as cancer and neurological disorders (9). Some of the proposed activities of apoA-I in these diseases have been suggested to be associated with its capacity to promote cellular cholesterol efflux and therefore modulate cellular cholesterol homeostasis and function (9).

Numerous rare point mutations have been identified in the human *APOA1* gene, often leading to reductions in HDL-C levels. Several of these mutations have also been associated with an elevated risk of CVD, increased overall mortality, as well as familial apoA-I amyloidosis (20, 21). One such mutation, the apoA-I[L178P] (also designated as L202P using the amino acid numbering of the entire translated protein) leads to low HDL-C levels, as well as increased carotid intima-media thickness and a 24-fold increase in CVD risk in heterozygous carriers (22). Previous studies from our lab showed that lipid-free apoA-I[L178P] displays major structural perturbations and thermodynamic destabilization (23). Furthermore, the folding defects of lipid-free apoA-I[L178P] were accompanied by a reduced capacity of the protein to promote ABCA1-mediated cholesterol efflux (23), a finding that could underlie the reduced HDL-C levels in carriers, as well as the increased CVD risk.

Malformed or misfolded proteins, caused even by very minor changes such as a single amino acid substitutions that severely alter protein structure and function, have been associated with several diseases. Small-molecule structure correctors that can reverse aggregation, abnormal intracellular localization or trafficking, and several functional defects of misfolded proteins hold promise for the treatment of diseases, including the transmembrane conductance regulator related to cystic fibrosis (24), the α-galactosidase A related to Fabry disease (25), and p53 in various cancers (26). Structure correctors are also being evaluated against the apoE, a protein bearing structural similarities to apoA-I (27), that is involved in the pathogenesis of Alzheimer’s disease. Screening of chemical libraries has resulted in the identification of small-molecule structure correctors that modulate the protein structure and rescue the apoE-associated neuropathology (28, 29).

In the current study, a library of marketed drugs (956 compounds) was screened against apoA-I[L178P] to identify molecules that can thermodynamically stabilize apoA-I and prevent mutant apoA-I from adopting its defective conformation. Screening was performed by the thermal shift assay (TSA) in the presence of the fluorescent dye SYPRO Orange. As an orthogonal assay, the monitoring of the change of fluorescence intensity of 8-anilinonaphthalene-1-sulfonic acid (ANS) upon its binding on hydrophobic sites on apoA-I was used. Selected compounds were further characterized for their capacity to ameliorate the structural and thermodynamic defects of apoA-I[L178P] and restore the capacity of the protein to promote ABCA1-mediated cholesterol efflux.

## Materials and methods

### Materials

Pfizer Licensed Compound Library (81 drug compounds supplied as predissolved DMSO solutions at a concentration of 10 mM) was purchased from Selleck Chemicals. Food and Drug Administration (FDA)-approved drugs screening library (875 FDA-approved drug compounds supplied as predissolved DMSO solutions at a concentration of 10 mM), atorvastatin (calcium salt), bexarotene, adapalene, and lovastatin were from Cayman Chemical. Sypro Orange and ANS probes were from Sigma-Aldrich. Strain BL21-Gold (DE3) of *Escherichia coli* was obtained from Stratagene. Ni^2+^-nitrilotriacetic acid (Ni-NTA) resin was purchased from Thermo Fisher Scientific. 4-[^14^C]Cholesterol (0.1 mCi/ml, specific activity 50 mCi/mmol) was obtained from American Radiolabeled Chemicals. Other reagents and consumables were purchased from Sigma-Aldrich, Biochrom AG, Lonza, Thermo Fisher Scientific, Bio-Rad, Roche, or other standard commercial sources.

### Production of WT apoA-I and apoA-I[L178P]

WT apoA-I and apoA-I[L178P] were produced using a bacterial expression system as described previously (23, 30). Briefly, *E. coli* BL21-Gold (DE3) cells, transformed with WT or mutant apoA-I–expressing vectors, were grown at 37°C until the absorbance of culture reached 0.6. At that point, protein expression induction was triggered by the addition of isopropyl β-D-1-thiogalactopyranoside (0.5 mM final concentration) in the bacterial cells and incubation for 2.5 h at 37°C.

All proteins were expressed in soluble form, fused with a thioredoxin tag, a 6× His-tag and a 3C-protease site at the fusion junction between the tags and apoA-I, and purified by Ni-NTA affinity chromatography (23, 30). The thioredoxin/His-tag was subsequently removed from apoA-I following treatment with a His-tagged 3C protease, and the released apoA-I was isolated by a second Ni-NTA resin affinity chromatography step in the flow through (23, 30). After purification, each apoA-I form was extensively dialyzed against 5 mM NH_4_HCO_3_, lyophilized, and stored at −80°C.

Before analyses, the lyophilized proteins were dissolved in 8 M guanidine hydrochloride in 50 mM sodium phosphate buffer, pH 7.4, and refolded by extensive dialysis against the same buffer. The samples were then centrifuged at 10.000 *g* for 10 min at 4°C, to remove any precipitated protein. Freshly refolded proteins were used for all analyses.

### Thermal shift assay

Thermal shift assay was performed as described previously (31, 32) with some modifications. Specifically, WT apoA-I or apoA-I[L178P], at a final concentration of 0.14 mg/ml (5 μM) in 50 mM sodium phosphate buffer, pH 7.4, were mixed with each library compound at a final concentration of 0.1 mM in wells of white 96-well plates. DMSO was added to control proteins (run in the absence of compounds) to match final DMSO concentration reached upon compound addition. Compounds alone, in the absence of protein, were also run in parallel. Sypro Orange dye (supplied as a 5,000× concentrate) was added to all mixtures at a final concentration of 20×. The total volume of mixtures was 20 μl. TSA measurements were performed using the high-resolution melting setting of LightCycler 96 instrument (Roche). The data of melting curves were exported using the Roche instrument software. The apparent melting temperature (Tm) is identified by plotting the first derivative of the fluorescence emission as a function of temperature (−dF/d*T*) and is represented as the lowest part of the curves.

### ANS fluorescence measurement

The fluorescence signal of ANS (final concentration 310 μM) following binding to WT apoA-I or apoA-I[L178P] [0.062 mg/ml (2.2 μM) in 50 mM sodium phosphate buffer, pH 7.4], in the absence or presence of 0.1 mM of library compounds, was measured by recording emission spectra from 425 to 600 nm at a 395 nm excitation wavelength, using an Infinite M200 microplate reader (Tecan), as described (23).

### ApoA-I α-helical content analysis

The far-UV CD spectra of lipid-free WT apoA-I and apoA-I[L178P] samples [0.1 mg/ml (3.6 μM) in 50 mM sodium phosphate buffer, pH 7.4], in the absence or presence of selected compounds at various concentrations, were recorded from 190 to 260 nm at 25°C, using a Jasco J-715 spectropolarimeter, as described before (23). Stock compound solutions were prepared in methanol. An appropriate volume of methanol was added to control proteins (run in the absence of compounds) to match the final methanol concentration reached upon compound addition. The helical content of apoA-I forms was calculated using the equation: % α-helix = (−[Θ]_222_ + 3,000)/(36,000 + 3,000) × 100 (33).

### Thermal denaturation experiments

To analyze the thermal denaturation profile of WT apoA-I and apoA-I[L178P] [0.1 mg/ml (3.6 μM) in 50 mM sodium phosphate buffer, pH 7.4], we recorded the change in molar ellipticity at 222 nm of lipid-free WT or mutant apoA-I forms, in the absence or presence of selected compounds at various concentrations, during gradual increase of temperature from 20°C to 80°C at a rate of 1°C/min, using a Jasco J-715 spectropolarimeter (23). Boltzmann simple sigmoidal model curve was used to fit the data by using the GraphPad Prism software, allowing the determination of the apparent Tm, as the midpoint of the thermal transition. In addition, the relative enthalpy change ΔH of the transition was determined as described before (34).

### Chemical denaturation experiments

To analyze the chemical denaturation profile of WT apoA-I and apoA-I[L178P] [0.1 mg/ml (3.6 μM) in 50 mM sodium phosphate buffer, pH 7.4], in the absence or presence of selected compounds, we monitored the change in wavelength of maximum intrinsic fluorescence of tryptophans by recording emission spectra from 310 to 420 nm using a 295 nm excitation wavelength, upon adding increasing amounts of 8.0 M guanidine hydrochloride, as described previously (23). Fluorescence measurements were carried out using a Quantamaster 4 fluorescence spectrometer (Photon Technology International). A Boltzmann simple sigmoidal model curve was used to fit the data using the GraphPad Prism software, allowing the determination of the midpoint of denaturation D_1/2_. The free energy difference between the native state and unfolded state of the protein in the absence of denaturant ΔG_D_° was determined using a linear extrapolation method, as described (34).

### Isothermal titration calorimetry

To quantitate drug-apoAI interactions we performed Isothermal Titration Calorimetry (ITC) experiments using a nano ITC instrument (TA instruments). Three hundred fifty microliters of an apoA-I[L187P] solution in 50 mM sodium phosphate buffer, pH 7.4, and at a concentration of 10 μΜ was degassed and put in the sample cell of the instrument. A solution of either atorvastatin (at 5 mM in 50 mM sodium phosphate buffer/10% (v/v) methanol) or bexarotene (at 2 mM in 50 mM sodium phosphate buffer/10% (v/v) methanol) was loaded onto the syringe, and sequential injections of 1 μl were performed into the protein solution under continuous stirring. Throughout the titration, heat changes were monitored using the nano ITC instrument, allowing for the observation of binding interactions between the drug and the protein. Control titrations using either mock injectants (same buffer composition without the drug) or injections of the drug into buffer were used to subtract effects not related to the drug-protein interaction. The corrected data were fit to a simple binding model using GraphPad Prism 8.0 to calculate EC_50_ values.

### Cell viability assay

Cell viability of J774 mouse macrophages, in the presence of selected compounds, was measured by the 3-(4,5-dimethylthiazol-2-yl)-2,5-diphenyltetrazolium bromide assay as described (35). Briefly, J774 macrophages were plated on 96-well plates at a density of 8 × 10^3^ cells/well in DMEM supplemented with 10% FBS and antibiotics. The next day, cell medium was removed and the cells were incubated, in DMEM supplemented with 0.2% BSA and antibiotics, for 48 h. Following this incubation period, cell medium was removed and the cells were incubated in the absence or presence of selected compounds at various concentrations, in DMEM supplemented with 0.2% BSA and antibiotics, for 4 h. At the end of the incubation period, media were aspirated and the cells were further incubated in DMEM/0.2% BSA-containing 0.65 mg/ml 3-(4,5-dimethylthiazol-2-yl)-2,5-diphenyltetrazolium bromide for 3 h at 37°C. Finally, the medium was removed, the dark blue formazan crystals formed by the cells were dissolved in DMSO, and absorbance was measured at 550 nm by an Infinite M200 plate reader (Tecan).

### Cholesterol efflux assay

ABCA1-dependent cholesterol efflux capacity of apoA-I forms, in the presence of selected compounds, was measured in J774 mouse macrophages, following induction with a cAMP analog that leads to increased expression of ABCA1 as described (23). Briefly, J774 mouse macrophages were labeled with 0.25 μCi/ml 4-[^14^C]-cholesterol for 24 h and then treated in the absence or presence of 0.3 mM cpt-cAMP [8-(4-chloro phenylthio)-cAMP] for 18 h. At the end of this treatment period, the cells were incubated with 1 μΜ lipid-free WT apoA-I or apoA-I[L178P], in the absence or presence of selected compounds at various concentrations, for 4 h. The radioactivity of cell media and cell lysates were determined by liquid scintillation counting. The [^14^C]-cholesterol efflux was expressed as the percentage of radioactivity released in the medium relative to total radioactivity in cells and medium. To calculate the ABCA1-mediated (net cpt-cAMP-dependent) cholesterol efflux, the cholesterol efflux of the untreated with cpt-cAMP cells was subtracted from the cholesterol efflux of the cells treated with cpt-cAMP.

## Results

### Screening for structure correctors of apoA-I[L178P] by the TSA

Our previous biophysical studies have shown that lipid-free apoA-I[L178P] presents major defects in protein structure and stability, whereas lipoprotein-associated apoA-I[L178P] displays milder conformational perturbations (23). Furthermore, lipid-free apoA-I[L178P] has reduced capacity to promote ABCA1-mediated cholesterol efflux (23). Cholesterol efflux via ABCA1, an important process for both HDL formation and atheroprotection, is proposed to be mediated mostly by lipid-free/lipid poor apoA-I and not by HDL-associated apoA-I (36, 37). Thus, the current studies were performed only with lipid-free WT apoA-I and apoA-I[L178P].

**Fig. 1:**
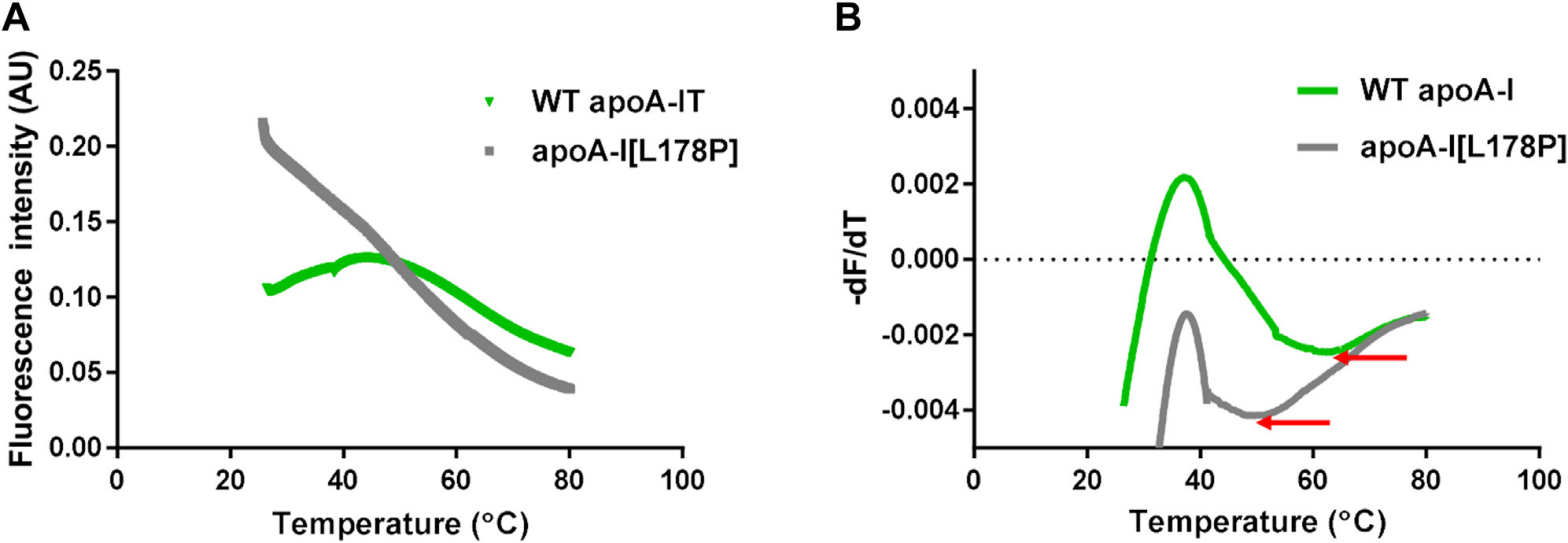
Typical thermal shift assay data and analysis. A: A typical thermal denaturation profile of recombinant WT apoA-I and mutant apoA-I[L178P] monitored by the change in fluorescence intensity of the dye SYPRO Orange, as the temperature of the sample is gradually increased. B: The Tm is identified by plotting the first derivative of the fluorescence emission as a function of temperature (−dF/dT). The Tm is calculated as the lowest part of the curve and is indicated by arrows. Tm, melting temperature.

To identify drugs that can correct the defective conformation of apoA-I[L178P], we carried out a TSA using recombinant WT apoA-I and apoA-I[L178P] in the absence and presence of drugs from two libraries, the Pfizer licensed compound library containing 81 drug compounds and the FDA-approved drugs screening library containing 875 FDA-approved drug compounds. The thermal denaturation profile of WT apoA-I and mutant apoA-I[L178P] is monitored by changes in fluorescence intensity, resulting from the interactions of proteins with the fluorescent dye SYPRO Orange, following the increase in temperature. Typical melting curves for WT and mutant apoA-I forms, in the absence of drugs, are shown in Fig. 1A. The thermal shift profile observed for WT apoA-I, which is a molten globule-like protein (38), is similar to the profile reported previously for molten globular proteins in TSA (31). By plotting the first derivative of the fluorescence emission as a function of temperature (−dF/d*T*), we can calculate the apparent Tm that corresponds to the lowest part of the curve (Fig. 1B). As shown in Fig. 1A, apoA-I[L178P], which has been found to display reduced thermodynamic stability (23), displays a distinct thermal denaturation profile compared to WT apoA-I. The Tm for apoA-I[L178P] is calculated to be 47°C, which is ∼18°C lower than the Tm for WT apoA-I, and is consistent with the Tm values that had been calculated previously for the thermal unfolding of WT and mutant apoA-I forms monitored by CD spectroscopy (23).

**Fig. 2:**
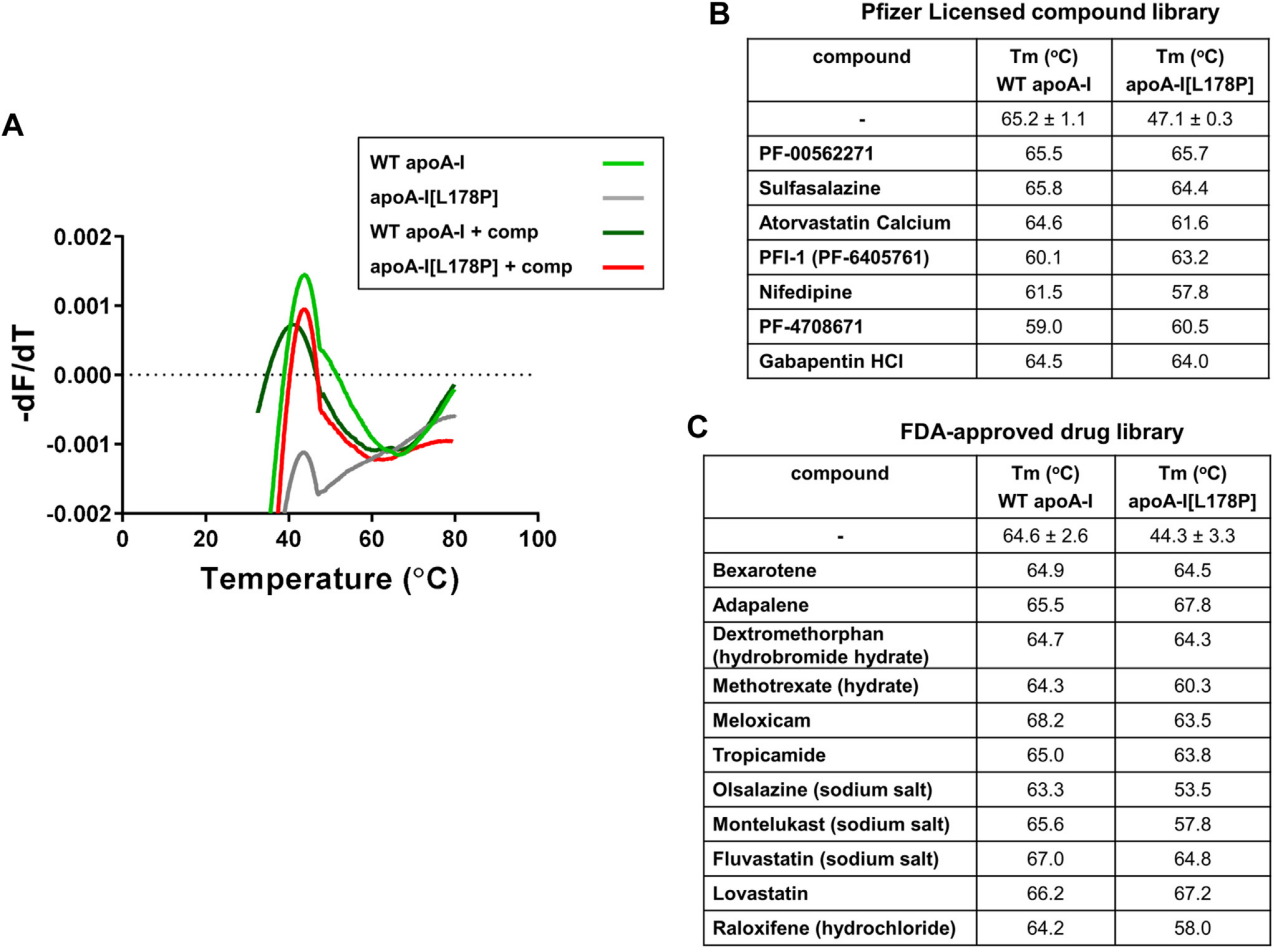
Screening of compounds from the Pfizer licensed compound library and the FDA-approved drug library, at a concentration of 0.1 mM, by TSA. A: Characteristic plots of the first derivative of the fluorescence emission of SYPRO Orange as a function of temperature (−dF/dT) for WT apoA-I and apoA-I[L178P] (5 μM) in the presence or absence of a potential structure corrector (0.1 mM). The compound induces the mutant protein to undergo a melting transition similar to that for WT apoA-I. Comp: gabapentin HCl. B, C: Tm values for WT and mutant apoA-I in the presence of selected molecules from the Pfizer licensed compound library (B) and the FDA-approved drug library (C). FDA, Food and Drug Administration; Tm, melting temperature.

Screening of compounds from the two drug libraries by TSA, at a concentration of 0.1 mM, resulted in the identification of seven compounds from the Pfizer licensed compound library and 11 compounds from the FDA-approved drugs screening library that induced a melting curve for apoA-I[L178P] to be similar to that of WT apoA-I (Fig. 2A, and supplemental Figs. S1 and S2). In addition, the Tm values for apoA-I[L178P] in the presence of these compounds are increased and in the majority of cases are similar to the value of Tm for WT apoA-I (Fig. 2B, C).

### ANS fluorescence measurement as a secondary assay for screening for structure correctors of apoA-I[L178P]

**Fig. 3:**
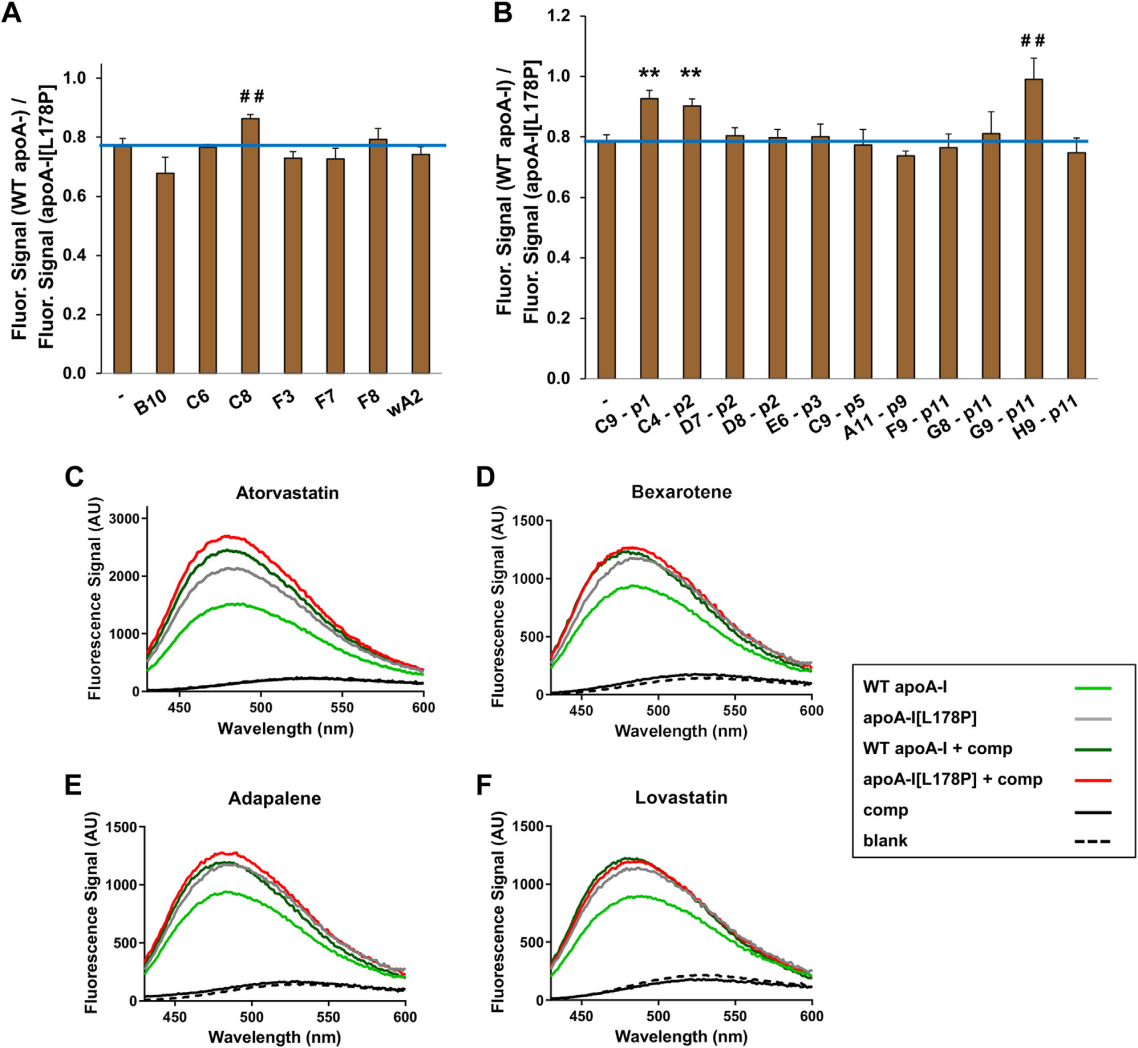
Binding of hydrophobic fluorescent probe ANS on WT apoA-I and apoA-I[L178P] in the presence of compounds from the Pfizer licensed compound library (A, C) and the FDA-approved drug library (B, D, E, and F). A, B: ratio of fluorescence signal of ANS binding to WT apoA-I against the fluorescence signal of ANS binding to apoA-I[L178P] (both proteins were at a concentration of 3.6 μM) in the absence or presence of selected compounds (0.1 mM) (compounds code names are described in supplemental Figs. S1H and S2L). ^##^*P* < 0.01 and ∗∗*P* < 0.005 versus proteins measured in the absence of compounds. C–F: ANS fluorescence spectra of WT or mutant apoA-I in the presence or absence of 0.1 mM atorvastatin (C8), bexarotene (C9-p1), adapalene (C4-p2), and lovastatin (G9-p11). ANS, 8-anilinonaphthalene-1-sulfonic acid; AU, arbitrary units; Comp, compound; FDA, Food and Drug Administration.

The 18 compounds, which were initially selected by the TSA screening, were further examined, at a concentration of 0.1 mM, for their capacity to bring apoA-I[L178P] to adopt similar structural properties with WT apoA-I by measuring the fluorescence intensity of the ANS amphipathic probe following its binding to the hydrophobic surface of apoA-I. In the absence of compounds, apoA-I[L178P] displays an increase in the solvent-accessible hydrophobic surface area in apoA-I as compared to WT apoA-I, likely due to the thermodynamic destabilization and partial denaturation or exposure of hydrophobic surfaces to the solvent, leading to higher ANS fluorescence signal (Fig. 3C–F), as reported before (23). Measurement of the fluorescence signal of ANS binding to WT apoA-I against the fluorescence signal of ANS binding to apoA-I[L178P], in the absence or presence of the 18 selected compounds, narrowed the potential structure correctors to 4. Specifically, atorvastatin, bexarotene, adapalene, and lovastatin resulted in a statistically significant increase in the ratio of fluorescence signals from WT and mutant apoA-I forms (Fig. 3A, B) by inducing apoA-I[L178P] to display similar ANS binding capacity with WT apoA-I (Fig. 3C–F).

### Concentration-dependent effect of selected compounds on the amelioration of defective apoA-I[L178P] structure monitored by the TSA

Subsequently, atorvastatin, bexarotene, adapalene, and lovastatin were examined at increasing concentrations, ranging from 0.01 to 0.5 mM, for their effect on the thermal profiles of WT apoA-I and mutant apoA-I[L178P] as assessed by the TSA (supplemental Figs. S3–S6). The analysis showed that the thermal denaturation profile of apoA-I[L178P] in the presence of atorvastatin at concentrations ≥0.1 mM is similar to that of WT apoA-I (supplemental Fig. S3). Bexarotene, at concentrations ≥ 0.05 mM, was shown to induce a concentration-dependent change of the thermal denaturation profile of apoA-I[L178P] (supplemental Fig. S4). Of note, it was observed that bexarotene at concentrations ≥0.1 mM and especially at the two higher concentrations used (0.2 and 0.5 mM) induces major alterations in the thermal denaturation profile of WT apoA-I. Similarly, adapalene at all concentrations used induced a concentration-dependent change of the thermal denaturation profile of apoA-I[L178P], but at the two higher concentrations of 0.2 and 0.5 mM induced major alterations in the thermal denaturation profile of WT apoA-I (supplemental Fig. S5). Finally, lovastatin did not show any concentration-dependent modulation of the thermal denaturation profile of apoA-I[L178P] (supplemental Fig. S6).

Collectively, atorvastatin at concentrations 0.1–0.5 mM, bexarotene at concentrations 0.05–0.1 mM, and adapalene at concentrations 0.01–0.1 mM seem to be capable of ameliorating the aberrant thermodynamic profile of apoA-I[L178P]. Therefore, these three compounds were selected to be further characterized for their capacity to correct conformational and functional defects of apoA-I[L178P].

### Effect of atorvastatin, bexarotene, and adapalene on J774 macrophages viability

**Fig. 4:**
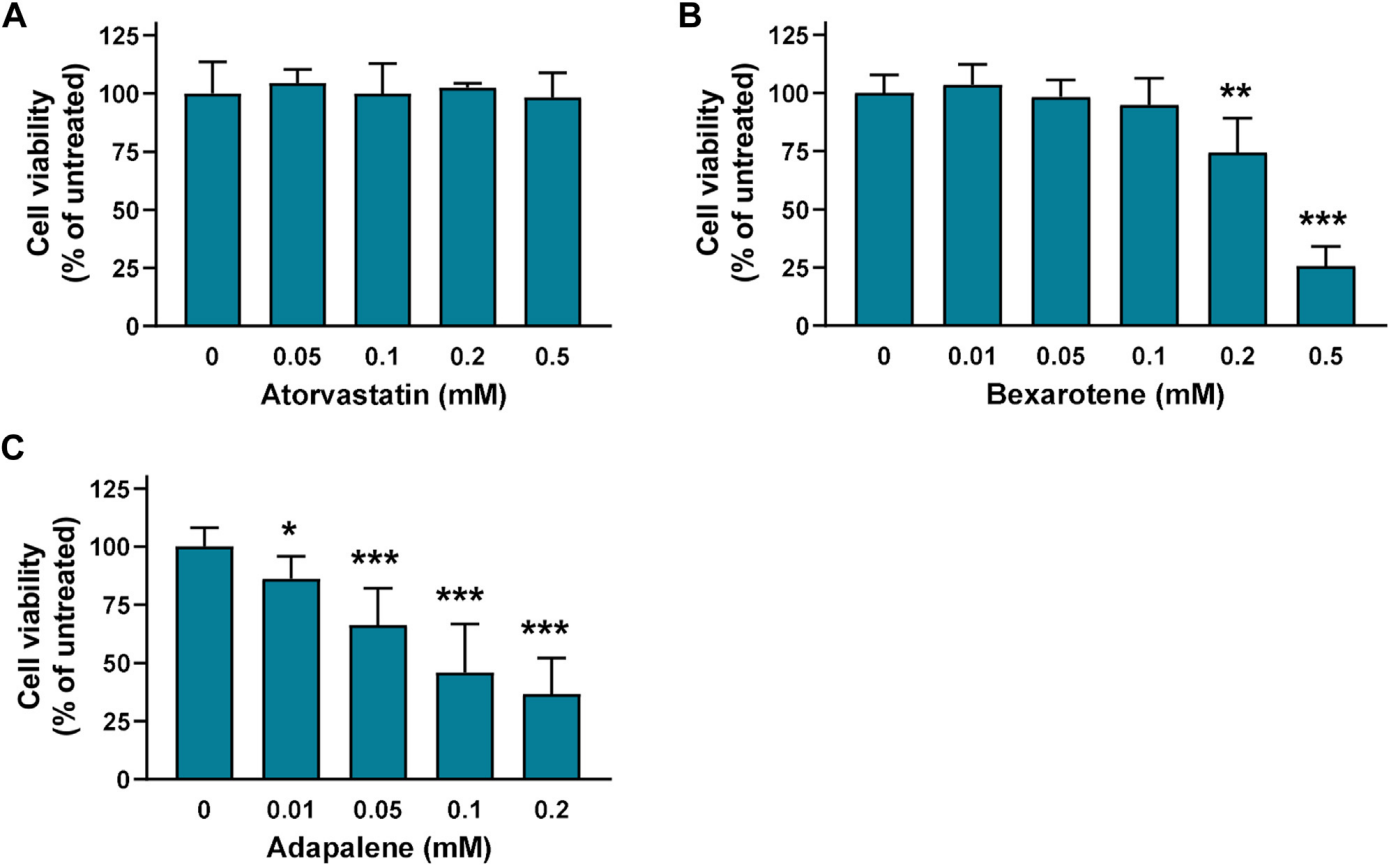
Effect of atorvastatin, bexarotene, and adapalene on J774 macrophages viability. Survival of J774 macrophages incubated with increasing concentrations of atorvastatin (A), bexarotene (B), and adapalene (C) for 4 h, as determined by a MTT assay. Cell viability is expressed as percent relative to the viability of untreated (control) cells set to 100%. Data are the means ± SD of three experiments performed in duplicate. ∗*P* < 0.05, ∗∗*P* < 0.005, and ∗∗∗*P* < 0.0001 versus untreated cells. MTT, 3-(4,5-dimethylthiazol-2-yl)-2,5-diphenyltetrazolium bromide.

Given that we aimed to identify molecules that can improve or restore the capacity of apoA-I[L178P] to promote ABCA1-cholesterol efflux from macrophages, before any further characterization of the effect of atorvastatin, bexarotene, and adapalene on structural and functional properties of the mutant apoA-I, we proceeded to examine their effect on J774 mouse macrophages viability. The analysis showed that atorvastatin did not affect cell viability at all concentrations tested (0.05–0.5 mM) (Fig. 4A). Bexarotene did not affect cell viability at concentrations 0.01–0.1 mM but induced a ∼25% reduction of cell viability at 0.2 mM and a severe reduction (∼75%) of cell viability at 0.5 mM (Fig. 4B). Adapalene displayed a severe reduction of viability of J774 mouse macrophages, showing cell toxicity even at the lower concentration used (0.01 mM) (Fig. 4C). Consequently, adapalene was not evaluated further.

### Evaluation of atorvastatin and bexarotene for their capacity to ameliorate defects on secondary structure and thermodynamic stability of apoA-I[L178P] assessed by CD and fluorescence spectroscopy

We proceeded to validate atorvastatin and bexarotene by examining their effect on WT and mutant apoA-I structure, following measurements of secondary structure by CD spectroscopy and thermodynamic stability versus temperature or chemical denaturants by CD and fluorescence spectroscopy, respectively. These biophysical techniques are extensively used in the literature for the characterization of the secondary structure and thermodynamic stability of apolipoproteins and specifically for characterizing the aberrant behavior of apoA-I[L178P] (23).

**Fig. 5:**
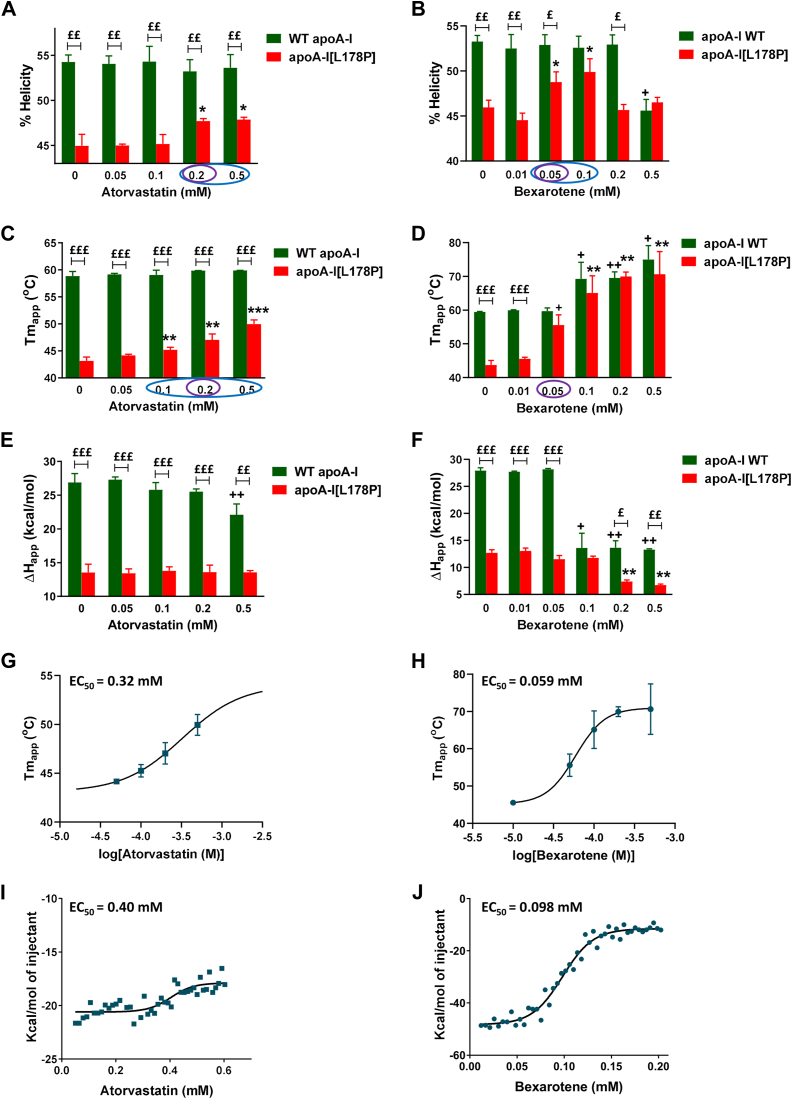
Effect of atorvastatin and bexarotene on secondary structure and thermodynamic stability parameters of WT apoA-I and apoA-I[L178P]. A, B: Percent α-helical content of WT apoA-I and apoA-I[L178P] (3.6 μM), in the presence of atorvastatin and bexarotene, calculated by CD spectroscopy. C–F: apparent Tm and ΔH values for the heating-induced transitions of WT and mutant apoA-I (3.6 μM), in the presence of atorvastatin and bexarotene, monitored by CD spectroscopy. G, H: Plots of apparent Tm values of apoA-I[L178P] against the concentration of atorvastatin and bexarotene. Solid line indicates fit to a simple binding model. Calculated EC_50_ is indicated. I, J: Isothermal titration calorimetry analysis of drug interaction with apoA-I[L178P] (10 μM). Plots show heat released from a solution of apoA-I[L178P] upon titration of either atorvastatin or bexarotene. Solid line indicates fit to a simple binding model. Calculated EC_50_ is indicated. Values represent the means ± SD (n = 3–4). ^+^*P* < 0.05 and ^++^*P* < 0.005 indicate comparisons for WT apoA-I in the presence and absence of compound; ∗*P* < 0.05, ∗∗*P* < 0.005, and ∗∗∗*P* < 0.0001 indicate comparisons for apoA-I[L178P] in the presence and absence of compound; ^£^*P* < 0.05, ^££^*P* < 0.005, and ^£££^*P* < 0.0001 indicate comparisons between the two apoA-I forms. Circles indicate the optimum concentrations of atorvastatin and bexarotene that improve the biophysical parameters for apoA-I[L178P] without affecting those for WT apoA-I. Tm, melting temperature.

We recorded the CD spectra of WT apoA-I and apoA-I[L178P] in the presence of increasing concentrations of atorvastatin (0.05–0.5 mM) (supplemental Fig. S7) and calculated the % α-helical content of each protein. The analysis showed that although the helicity of apoA-I[L178P] remained lower than that of WT apoA-I in the presence of any concentration of atorvastatin, there was a small but statistically significant increase in the presence of 0.2 and 0.5 mM atorvastatin (Fig. 5A). In addition, we examined the thermodynamic stability of apoA-I forms, in the presence of increasing concentrations of atorvastatin, by following the CD signal at 222 nm while the protein was gradually unfolded by increasing temperature (supplemental Fig. S8). Analysis of thermal denaturation profiles and calculation of the apparent Tm midpoint showed again that although the Tm values of thermal denaturation of apoA-I[L178P] remained lower than that of WT apoA-I in the presence of all concentrations of atorvastatin, there was a concentration-dependent increase of Tm for apoA-I[L178P] (Fig. 5C). In contrast, atorvastatin did not affect the apparent enthalpy change of the transition ΔH of apoA-I[L178P] at any concentration used (Fig. 5E). Additionally, there was no effect of atorvastatin, at any concentration used, on the helical content and Tm value of WT apoA-I (Fig. 5A, C), but at the highest concentration used (0.5 mM), we observed a thermodynamic destabilization for WT apoA-I as indicated by the thermal denaturation curve and a reduction in ΔH value (supplemental Fig. S8D and Fig. 5E).

The same set of biophysical analyses were also performed for WT apoA-I and apoA-I[L178P] in the presence of increasing concentrations of bexarotene (0.01–0.5 mM). We found that bexarotene induces an increase of helicity of apoA-I[L178P] at concentrations 0.05 and 0.1 mM (Fig. 5B). Of note, in the presence of 0.1 mM bexarotene, the percent α-helical content of apoA-I[L178P] was similar to that of WT apoA-I (supplemental Fig. S9 and Fig. 5B). However, in the presence of higher concentrations of bexarotene, the helicity of apoA-I[L178P] was reduced, while at the highest concentration used (0.5 mM) the helicity of WT apoA-I was also reduced (Fig. 5B). Thermal denaturation analysis showed that at concentrations ≥0.05 mM of bexarotene the Tm of thermal denaturation transition of apoA-I[L178P] is increased (Fig. 5D). Interestingly, in the presence of 0.05 mM bexarotene, the Tm for apoA-I[L178P] was similar to that for WT apoA-I, while bexarotene did not disturb the conformation of WT apoA-I. However, higher concentrations of bexarotene resulted in modulation of the proper conformation of WT apoA-I, as indicated by the thermal denaturation profiles (supplemental Fig. S10C–E), as well as the increase in Tm and decrease in ΔH values (Fig. 5D, F).

Collectively, atorvastatin at concentrations 0.1–0.5 mM and bexarotene at concentrations 0.05–0.1 mM appear to induce apoA-I[L178P] to adopt a conformation more similar to that of WT apoA-I. The optimum concentration of Atorvastatin is around 0.2 mM and of Bexarotene around 0.05 mM since at these concentrations the compounds do not seem to disturb the conformation of WT apoA-I. Plotting of Tm values of apoA-I[L178P] against the concentration of atorvastatin and bexarotene showed that the EC_50_ value of drugs was 0.32 mM for atorvastatin and 0.059 mM for bexarotene (Fig. 5G, H).

To confirm the calculated EC_50_ values by an orthogonal assay, we used ITC (Fig. 5I, J). Sequential injections of either atorvastatin or bexarotene to a solution of apoA-I[L178P] were performed in a nano ITC instrument while measuring the heat changes associated with the binding process. Data were corrected for solvent effects by performing blank titrations. Experimental data were fit to a simple binding model, resulting to calculated EC_50_ values of 0.40 mM and 0.098 mM for atorvastatin and bexarotene respectively, which are in good agreement with the values calculated from thermal denaturation experiment, providing additional validation for the drug-protein interactions.

**Fig. 6:**
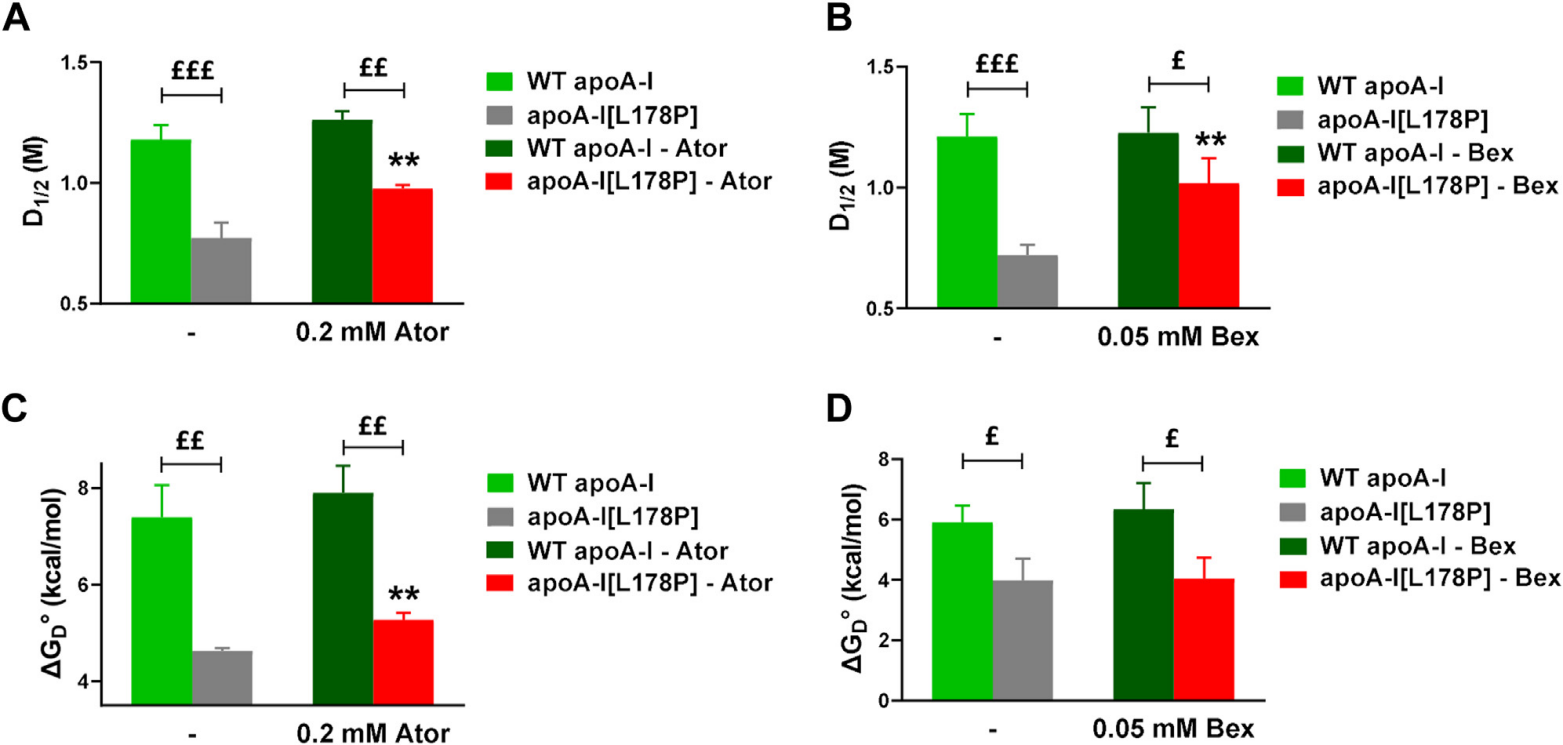
Effect of atorvastatin and bexarotene on chemical denaturation of WT apoA-I and apoA-I[L178P]. D_1/2_ (A, B) and ΔG_D_° (C, D) values for the denaturant-induced transitions of WT apoA-I and apoA-I[L178P] (3.6 μM), in the presence of atorvastatin and bexarotene, monitored by fluorescence spectroscopy. Values represent the means ± SD (n = 3–4). ∗∗*P* < 0.005 indicate comparisons for apoA-I[L178P] in the presence and absence of compound; ^£^*P* < 0.05, ^££^*P* <0.005, and ^£££^*P* < 0.0001 indicate comparisons between the two apoA-I forms. Ator, atorvastatin; Bex: bexarotene.

Atorvastatin (0.2 mM) and bexarotene (0.05 mM) were also shown to result in an increase of the midpoint of denaturation D_1/2_ of apoA-I[L178P], as assessed by chemical denaturation analysis of WT and mutant apoA-I forms (supplemental Fig. S11 and Fig. 6A, B). Additionally, atorvastatin induces a small but statistically significant increase in the free energy change ΔG_D_° of apoA-I[L178P] (Fig. 6C).

### Evaluation of atorvastatin and bexarotene for their capacity to amend the defective capacity of apoA-I[L178P] to promote ABCA1-mediated cholesterol efflux

**Fig. 7:**
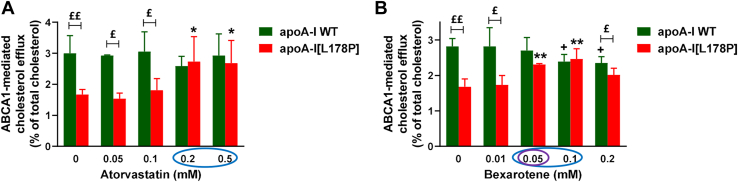
Effect of atorvastatin and bexarotene on the capacity of WT apoA-I and apoA-I[L178P] to promote ABCA1-mediated cholesterol efflux from J774 macrophages. J774 mouse macrophages were labeled with [^14^C]-cholesterol, treated in the presence or absence of cpt-cAMP, and incubated with WT or mutant apoA-I forms (1 μM), in the presence or absence of increasing concentrations of atorvastatin (A) or bexarotene (B), for 4 h. The net ABCA1-mediated (cpt-cAMP dependent) cholesterol efflux is calculated as the difference in cholesterol efflux between cpt-cAMP–treated cells and nontreated cells. Values are the means ± SD for three independent experiments performed in duplicate or triplicate. ^+^*P* < 0.05 indicate comparisons for WT apoA-I in the presence and absence of compound; ∗*P* < 0.05 and ∗∗*P* < 0.005 indicate comparisons for apoA-I[L178P] in the presence and absence of compound; ^£^*P* < 0.05 and ^££^*P* < 0.005 indicate comparisons between the two apoA-I forms. Circles indicate the optimum concentrations of atorvastatin and bexarotene that restore the cholesterol efflux capacity of apoA-I[L178P], without affecting or inducing only a small reduction in the cholesterol efflux capacity of WT apoA-I.

Given the findings on the capacity of atorvastatin and bexarotene to ameliorate the structural defects of apoA-I[L178P], we proceeded to examine the effect of these two compounds on the capacity of the mutant protein to promote ABCA1-mediated cholesterol efflux, which was found previously to be ∼35% reduced as compared to the capacity of WT apoA-I (23). As shown in Fig. 7A, atorvastatin at concentrations of 0.2 and 0.5 mM fully restored the capacity of apoA-I[L178P] to promote ABCA1-mediated cholesterol efflux. Bexarotene at concentrations of 0.05 and 0.1 mM also fully restored the capacity of apoA-I[L178P] to promote ABCA1-mediated cholesterol efflux (Fig. 7B). However, bexarotene at a concentration of 0.1 mM induced a small, but statistically significant, reduction of the capacity of WT apoA-I for ABCA1-mediated efflux, while at the higher concentration of 0.2 mM bexarotene reduced the cholesterol efflux capacities of both WT-apoA-I and apoA-I[L178P] (Fig. 7B). Therefore, the cholesterol efflux analysis showed that the optimum concentrations of the drugs as functional correctors are 0.2–0.5 mM for atorvastatin and 0.05 mM for bexarotene. Collectively, our biophysical and cell-based assays showed that atorvastatin and bexarotene, when used at optimal concentrations, can amend both the structural and functional defects of apoA-I[L178P].

### Molecular docking suggests a possible mechanism for apoA-I stabilization by atorvastatin and bexarotene

**Fig. 8:**
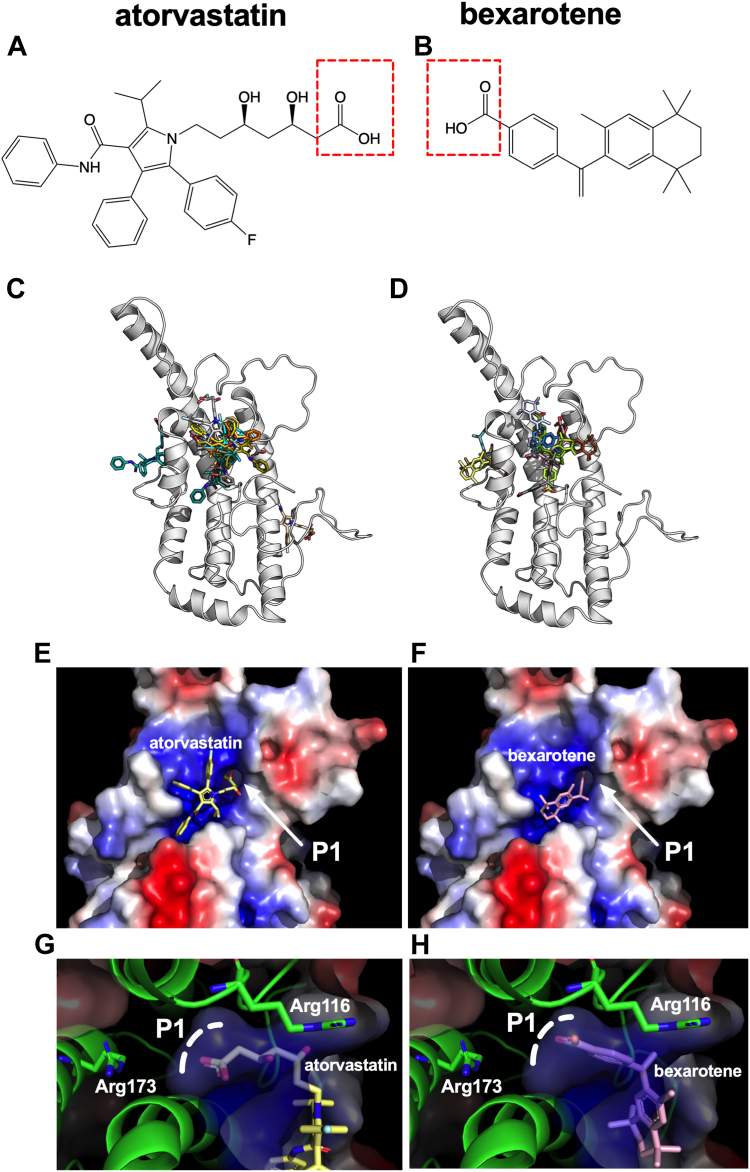
Molecular docking of atorvastatin and bexarotene onto the apoA-I structure. Panels (A, B): Chemical structures of atorvastatin and bexarotene. Red boxes highlight acidic groups in the structures. Panels (C, D): Schematic representation of the consensus structure of apoA-I, shown in cartoon representation, with the 10 best docked models of atorvastatin (panel C) and bexarotene (panel D). Most docked structures cluster around a single area. Panels E and F: ApoA-I model shown in surface representation and colored by calculated electrostatic potential (red = negative, blue = positive, and white = neutral), highlighting the position of the best model of atorvastatin and bexarotene (Table 1), as well as the location of the “P1” pocket. Panels (G, H): Close-up view of the P1 pocket of apoA-I where the best docked models for both drugs were found to make electrostatic interactions by the hydroxy pentanoic acid group of atorvastatin and the benzoic acid group of bexarotene. ApoA-I is shown in semi-transparent surface representation colored by electrostatic potential as in Panels (E, F). ApoA-I residues Arg173 and Arg116 that help shape the pocket are shown as green sticks. The base of the P1 pocket is indicated by a dotted curved line.

To gain insight into the molecular mechanisms that could contribute to apoA-I stabilization and structure correction by atorvastatin and bexarotene, we performed molecular docking of the drugs onto the apoA-I structure using the HDOCK server (39). Since no full-length experimental structure of apoA-I has been reported, we used the consensus model of human apoA-I in its monomeric and lipid-free state as reported by Melchior *et al.* (4). The structures of atorvastatin and bexarotene were obtained from PubChem (40) (Fig. 8A, B) and docked to the full-length apoA-I consensus model. The best 10 interaction models as determined by their docking score are shown in Fig. 8C, D, for atorvastatin and bexarotene, respectively (docking parameters are shown in Table 1). The majority of the models suggested that both atorvastatin and bexarotene target the same cavity in apoA-I, a cavity formed between helix 1/2 and helix 5 with the participation of the extended loop connecting helix 5 and helix 6. This cavity has a strong positive electrostatic potential and forms a deep pocket (indicated as P1 in Fig. 8E–Η) that can accommodate the hydroxy pentanoic acid group of atorvastatin and the benzoic acid group of bexarotene by forming electrostatic interactions with the nearby Arg173 and Arg116, as well as hydrophobic van der Waals interactions with the hydrophobic base of Arg116 (Fig. 8G, H). The extensive interactions of both drugs with this cavity of apoA-I could contribute to the thermodynamic stabilization of the molecule. This would be especially important for the L178P variant which would be normally destabilized due to the introduction of the helix-breaker proline residue in the middle of a major helix of the protein (helix 5). The common binding pose of both drugs indicated by the docking calculations suggests that targeting this cavity in apoA-I by small molecules may be a valid strategy for structure correctors aiming to rescue disease phenotypes related to apoA-I thermodynamic destabilization. Further structural analysis will be necessary to confirm or disprove this mechanism.

**Table 1:** Calculated parameters by HDOCK for the 10 best binding models for each compound

Compound	Rank	1	2	3	4	5	6	7	8	9	10
Atorvastatin	Docking Score	−166.71	−163.44	−163.27	−149.52	−145.38	−141.74	−140.54	−139.6	−139.53	−137.43
	Confidence Score	0.5828	0.5668	0.566	0.4976	0.4769	0.4588	0.4528	0.4482	0.4478	0.4375
	Ligand rmsd (Å)	69.53	69.63	72.61	70.05	71.73	68.37	68.63	68.46	73.65	43.19
Bexarotene	Docking Score	−148.82	−121.73	−118.19	−115.01	−112.47	−112.45	−112.35	−111.84	−111.67	−111.53
	Confidence Score	0.4941	0.3623	0.3461	0.3319	0.3207	0.3206	0.3202	0.318	0.3172	0.3166
	Ligand rmsd (Å)	67.1	73.81	69.89	68.1	73.86	72.84	71.86	70.64	69.66	65.16

The docking score is calculated by an iterative scoring function and a more negative number indicates a more possible minding model. The confidence score, is a docking score-dependent parameter, which indicates the binding likeliness of the two-molecule interaction. Higher confidence scores indicate a more likely model. The ligand RMSDs are calculated by comparing the ligands in the docking models with the input or modeled structures.

## Discussion

This study was undertaken to identify small molecules that have the ability to modulate the structure and function of a pathogenic apoA-I variant, apoA-I[L178P]. Lipid-free apoA-I displays a high degree of conformational plasticity (41) and natural occurring point mutations, such as the L178P, have been shown to induce local but also global effects on the structure of protein (23, 42). Lipid-free apoA-I has a direct physiologically relevant role since it promotes ABCA1-mediated efflux of phospholipids and cholesterol, a step that is critical both for de novo HDL biogenesis by hepatocytes and for atheroprotection by intimal macrophages (10, 11). Previous structure-function relationship studies of naturally occurring and bioengineered apoA-I mutants have indicated that the conformation of apoA-I may affect its functional properties and underlie its role in HDL formation and/or atheroprotection (23, 30, 43). Therefore, structure correctors could be useful for the amelioration of defective apoA-I functions and prevention of apoA-I–related pathogenic conditions.

Our analyses showed that two drugs approved by the U.S. FDA, atorvastatin which is a cholesterol-lowering drug and bexarotene which is an anticancer drug, have the capacity to amend both structural and functional disturbances of apoA-I[L178P]. Interestingly, it was observed that these two compounds at concentrations that induce apoA-I[L178P] to increase its α-helical content also result in the restoration of the capacity of mutant apoA-I to induce ABCA1-mediated cholesterol efflux. Sufficient α-helical content and appropriate conformation of apoA-I have been shown previously to be necessary for the binding of apoA-I with lipids and efficient cholesterol efflux (44, 45).

While the exact mechanism employed by atorvastatin and bexarotene for stabilizing apoA-I will require further structural studies, docking calculations suggested that they both target the same cavity in the apoA-I structure by a combination of hydrophobic and electrostatic interactions and utilize a common binding motif that buries a hydrophobic group with an acidic “point” (the hydroxy pentanoic acid group of atorvastatin and the benzoic acid group of bexarotene, Fig. 8A, B) in a pocket that has a highly positive electrostatic potential (Fig. 8G, H). The common theme utilized by two chemically distinct compounds increases our confidence that the proposed mechanism is valid. Furthermore, this cavity is formed only in the well-folded apoA-I, consisting of structural features that only come together when apoA-I assumes its correct 3D structure, which could underlie the structural and thermodynamic stabilization of the protein. Further structure-activity optimization of these compounds will be necessary to develop derivatives that correct the structure of defective apoA-I variants at lower compound concentrations and with higher effectiveness.

A limitation of the current study is that the identified compounds, despite being commercially available drugs, could not realistically be administered as apoA-I structure correctors for pharmaceutical interventions due to their low potency, which would necessitate unrealistic high doses. In addition, our analyses indicated that exceeding an optimum concentration for atorvastatin and bexarotene results in adverse effects on apoA-I structure, suggesting possible additional interactions of the compounds with the protein when used at very high concentrations. One additional limitation of our approach is that our structural interpretations and insights are based on a model of apoA-I, and not an experimentally determined, high-resolution structure of apoA-I, which has not been achieved yet. While this model is currently the best option available, its use may carry caveats and thus our structural insights should be interpreted with caution. Besides these limitations, however, these two drugs constitute novel leads that can be further optimized by medicinal chemistry campaigns to enhance potency and effectiveness, potentially leading to compounds of pharmaceutical usefulness.

Structural and functional modulation by small molecules has been also shown for apoE, which is a protein with similar structural and functional properties to apoA-I. ApoE contains several amphipathic α-helical regions organized into two structural domains, is capable to bind lipids, promotes cholesterol efflux, and can form HDL-like particles (46, 47). Human apoE has three common isoforms (apoE2, apoE3, and apoE4) that differ at positions 112 or 158 (48). Single amino acid substitutions of these positions give rise to differences in the tertiary protein structure and function of apoE variants, as well as their role in disease, with apoE4 being the major risk factor for Alzheimer’s disease (46, 48). Compound library screening analyses and subsequent structural and functional characterizations identified small-molecule–based structure correctors that induce proper apoE4 folding and prevent functions of apoE4 that are related to Alzheimer’s disease pathogenesis (28, 29). Therefore, those and our studies indicate that the modulation of the structure and function of amphipathic apolipoproteins by small molecules could be a tractable approach for correcting aberrant structural properties related to disease.

Overall, our results serve as a proof of concept that small molecules can ameliorate defective apoA-I structure and function. The identified compounds can form the basis for the development of improved compounds that could, at pharmacologically relevant concentrations, induce mutant apoA-I variants to adopt a similar to WT apoA-I structure and function. Structure correctors could represent a novel therapeutic approach for treating apoA-I–related diseases, such as atherosclerotic cardiovascular disease and familial apoA-I amyloidosis (20, 21).

## Data availability

All data are contained within the manuscript and Supporting information.

## Supplemental data

This article contains supplemental data.

## Conflict of interest

The authors declare that they have no conflicts of interest with the contents of this article.
