# Supplementary material for: Library screening identifies commercial drugs as potential structure correctors of abnormal apolipoprotein A-I
Source: J Lipid Res. 2024 Apr 17;65(5):100543. doi: 10.1016/j.jlr.2024.100543 (PMC11106541; doi:10.1016/j.jlr.2024.100543)
Supplement: Supplemental Figures S1–S11 [file mmc1.docx]

**Library screening identifies commercial drugs as potential structure correctors of abnormal apolipoprotein A-I**

Christina Gkolfinopoulou^1^, Angeliki Bourtsala^1^, Daphne Georgiadou^1^, Anastasia-Georgia Dedemadi^1,2^, Efstratios Stratikos^2^ and Angeliki Chroni^1*^

*^1^Institute of Biosciences and Applications, National Center for Scientific Research “Demokritos”, Agia Paraskevi, Athens, Greece*

*^2^Department of Chemistry, National and Kapodistrian University of Athens, Zografou, Athens, Greece*

**
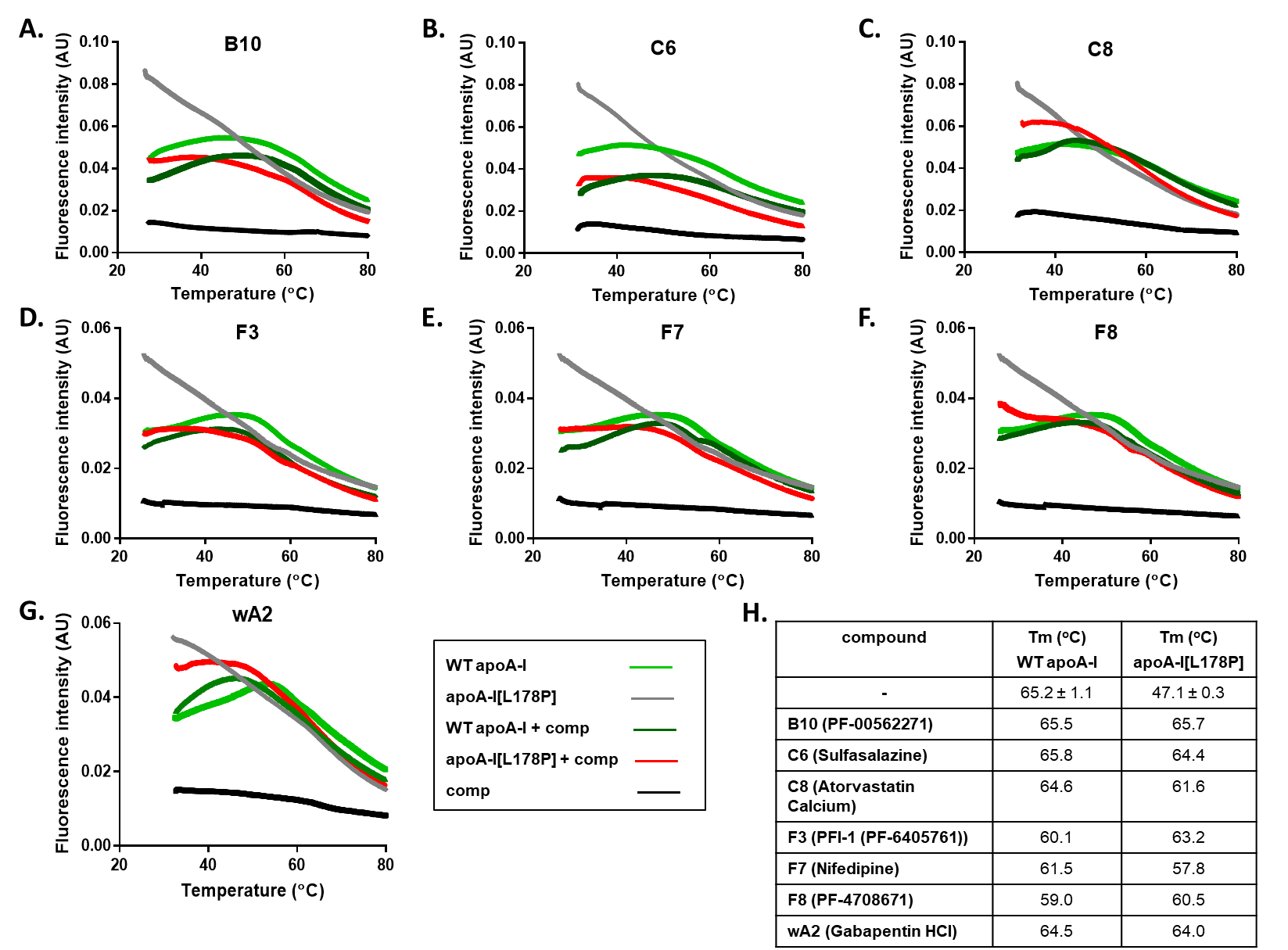
**

**Figure S1. Thermal denaturation profiles of WT apoA-I and apoA-I[L178P], in the presence of compounds from the Pfizer Licensed compound library, monitored by the thermal shift assay.** (A-G) Profiles for WT and mutant apoA-I (5 µM) in the absence and presence of molecules (0.1 mM) identified to induce the mutant protein to undergo a melting transition similar to that for WT apoA-I. (H) T_m_ values for WT and mutant apoA-I in the presence of selected molecules. Comp: compound.

**
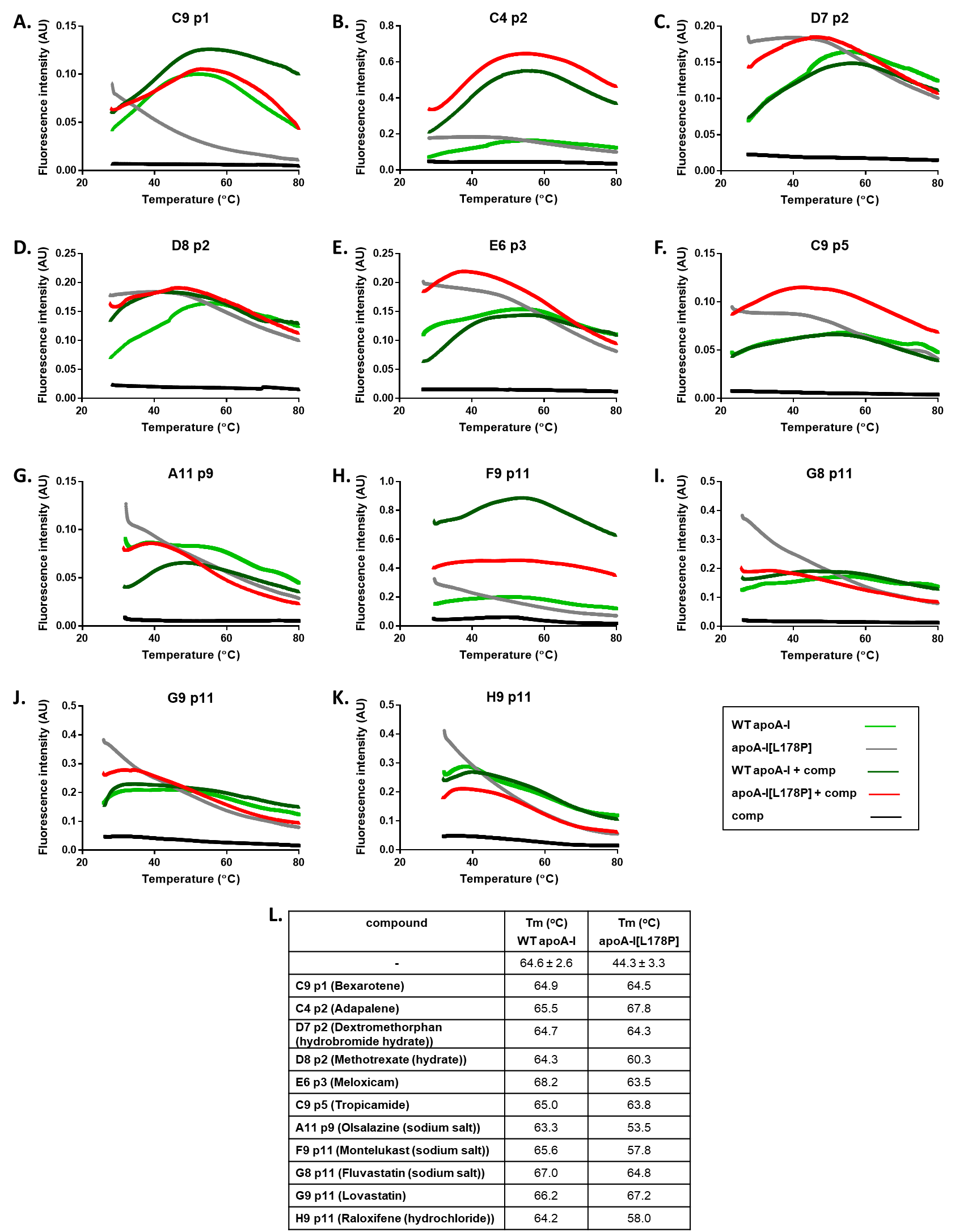
**

**Figure S2. Thermal denaturation profiles of WT apoA-I and apoA-I[L178P], in the presence of compounds from the FDA-approved drug library, monitored by the thermal shift assay.** (A-K) Profiles for WT and mutant apoA-I (5 µM) in the absence and presence of molecules (0.1 mM) identified to induce the mutant protein to undergo a melting transition similar to that for WT apoA-I. (L) T_m_ values for WT and mutant apoA-I in the presence of selected molecules. Comp: compound.

**
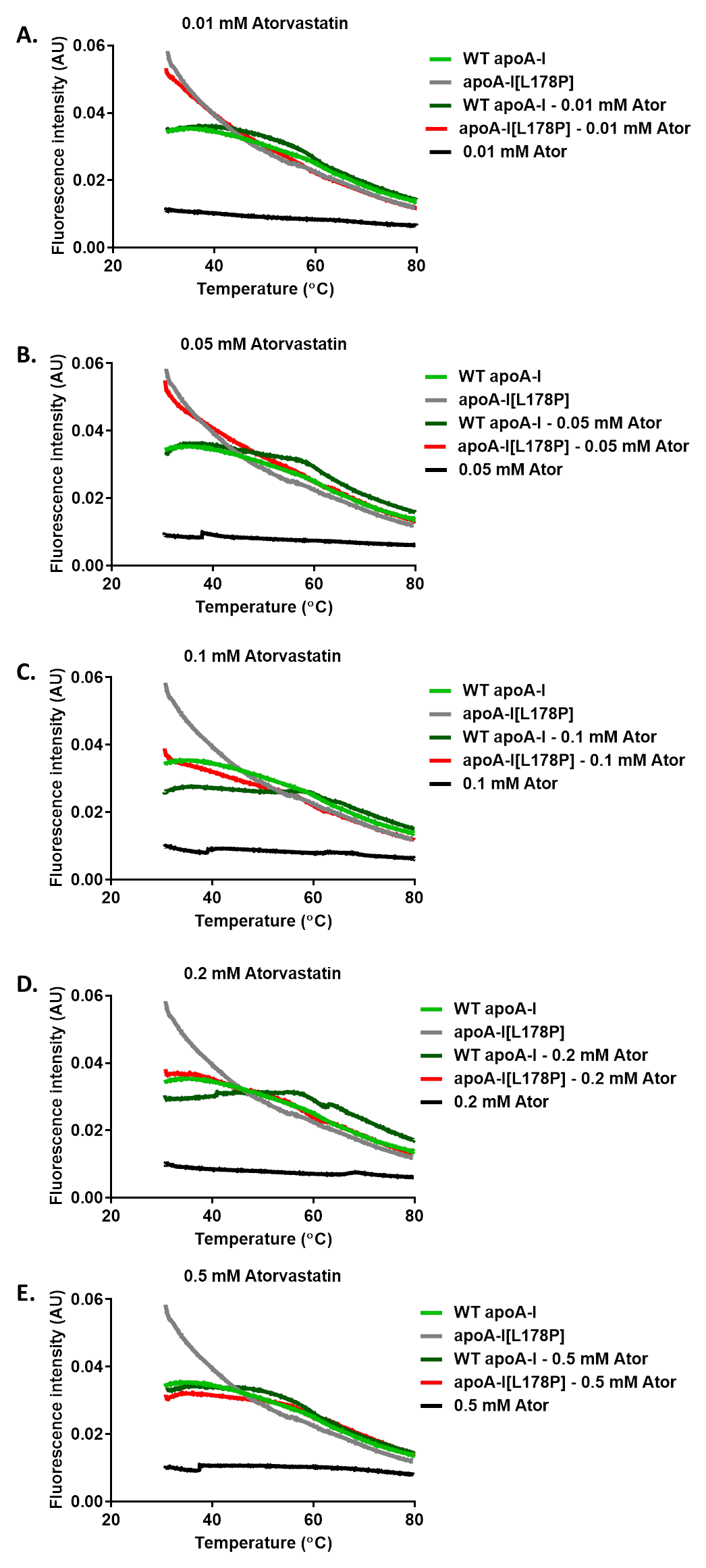
**

**Figure S3. Effect of Atorvastatin at increasing concentrations on thermal denaturation profiles of WT apoA-I and apoA-I[L178P] (5 µM), monitored by the thermal shift assay.**


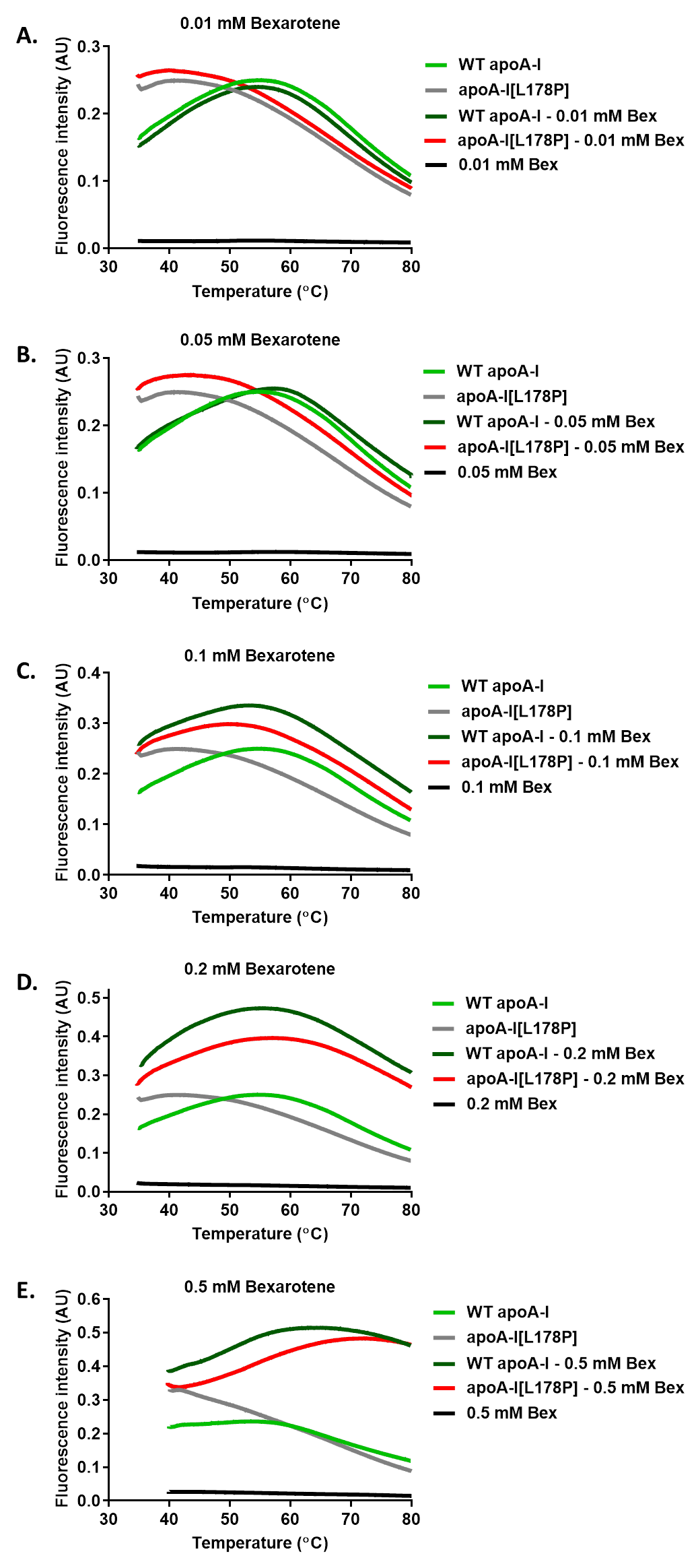


**Figure S4. Effect of Bexarotene at increasing concentrations on thermal denaturation profiles of WT apoA-I and apoA-I[L178P] (5 µM), monitored by the thermal shift assay.**


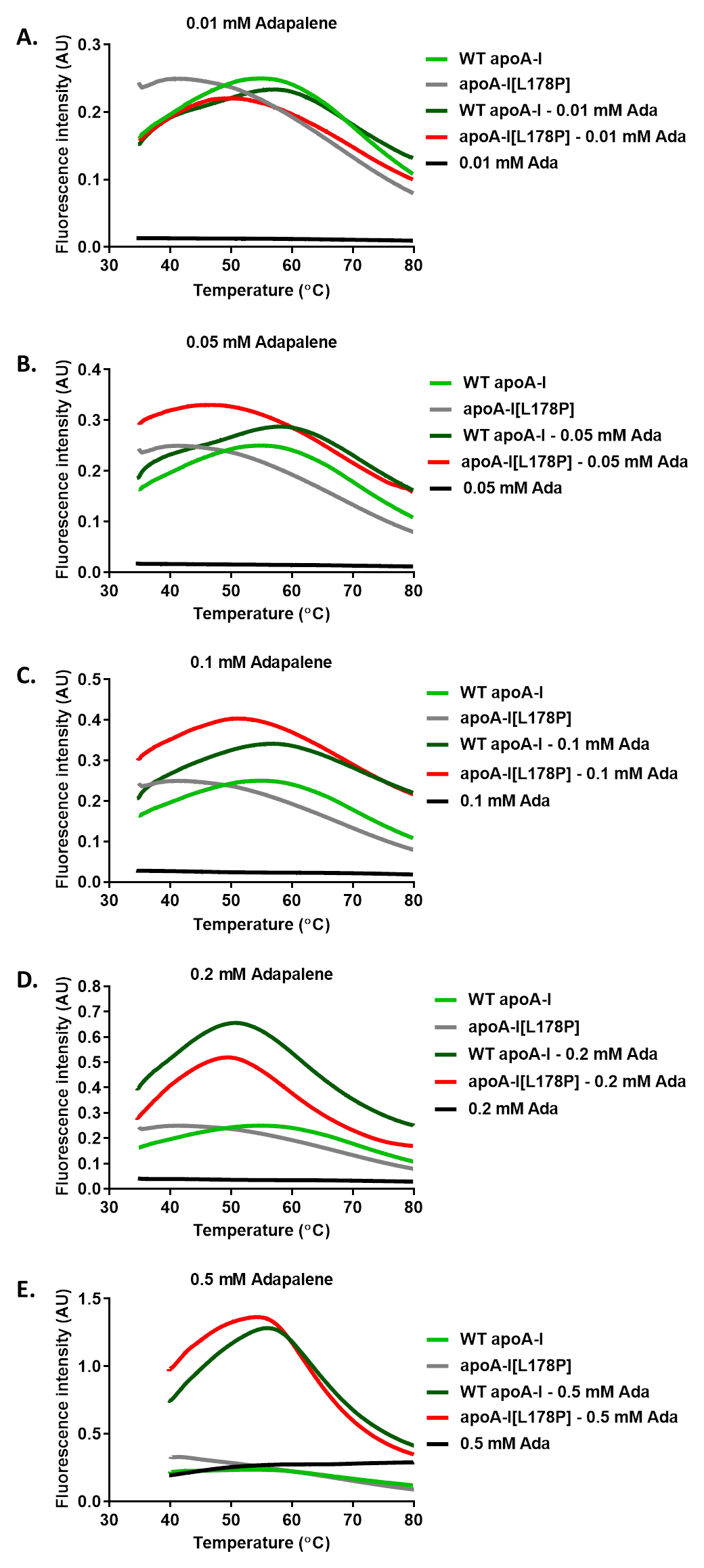


**Figure S5. Effect of Adapalene at increasing concentrations on thermal denaturation profiles of WT apoA-I and apoA-I[L178P] (5 µM), monitored by the thermal shift assay.**


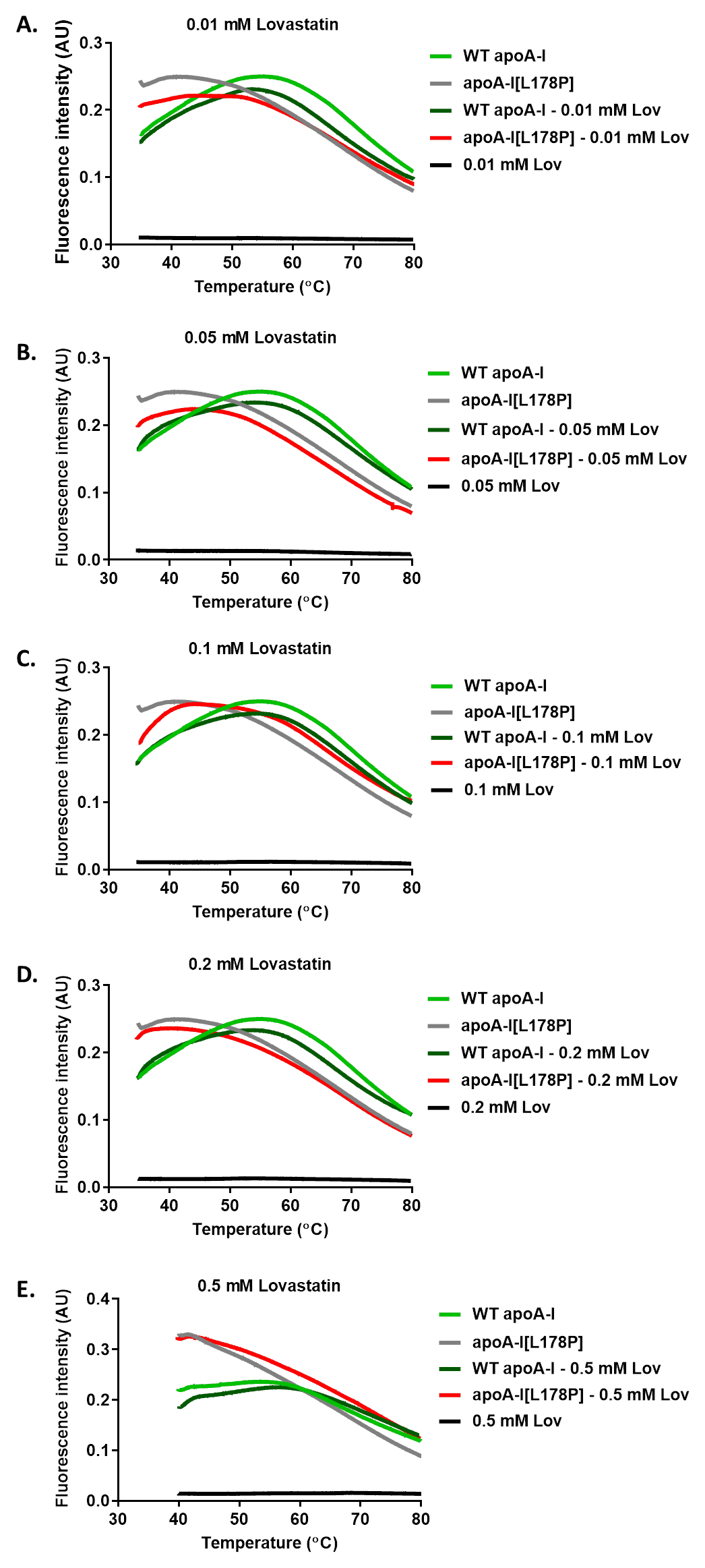


**Figure S6. Effect of Lovastatin at increasing concentrations on thermal denaturation profiles of WT apoA-I and apoA-I[L178P] (5 µM), monitored by the thermal shift assay.**


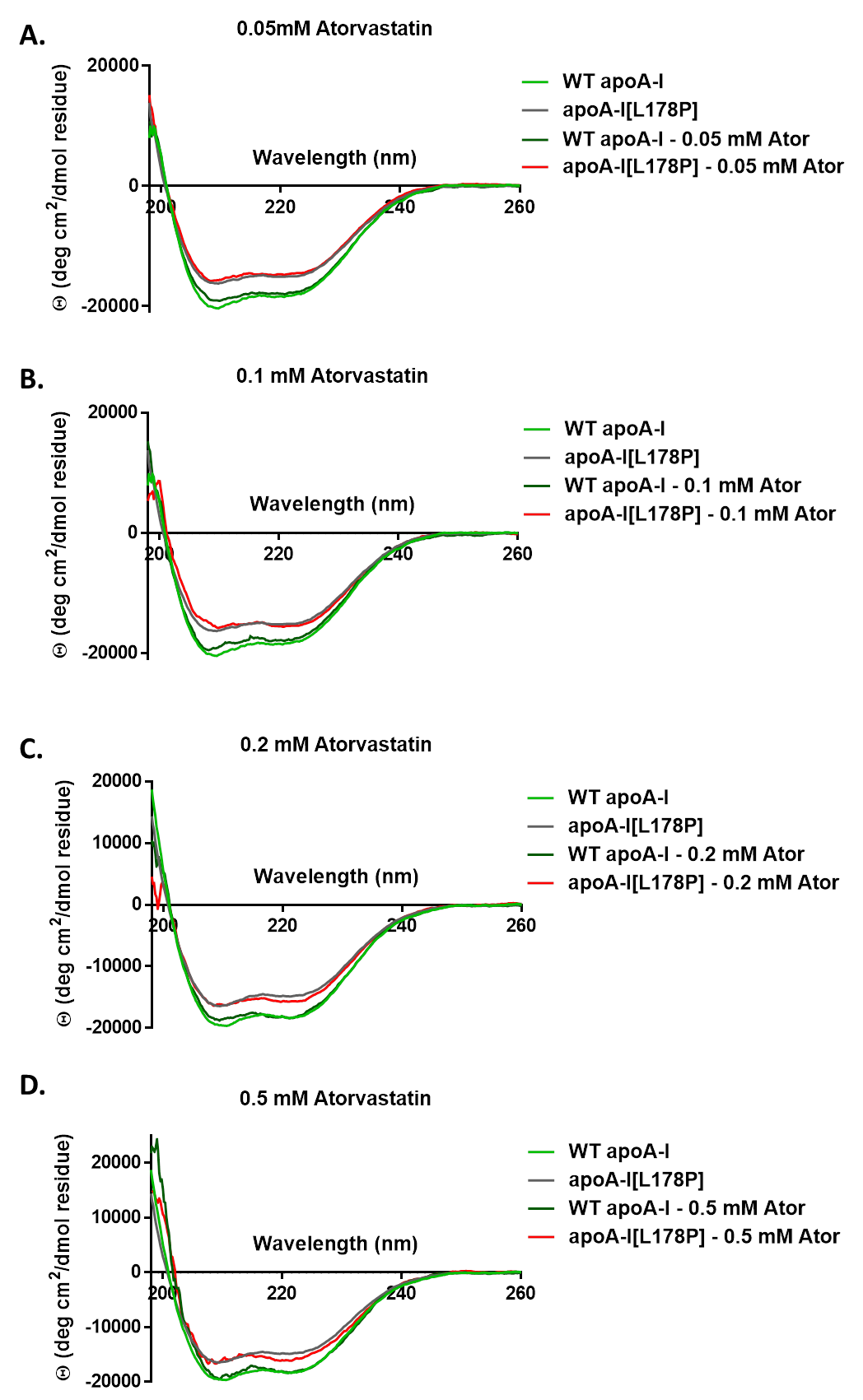


**Figure S7. Effect of Atorvastatin, at increasing concentrations, on the far-UV CD spectra of WT apoA-I and apoA-I[L178P] (3.6 µM).**


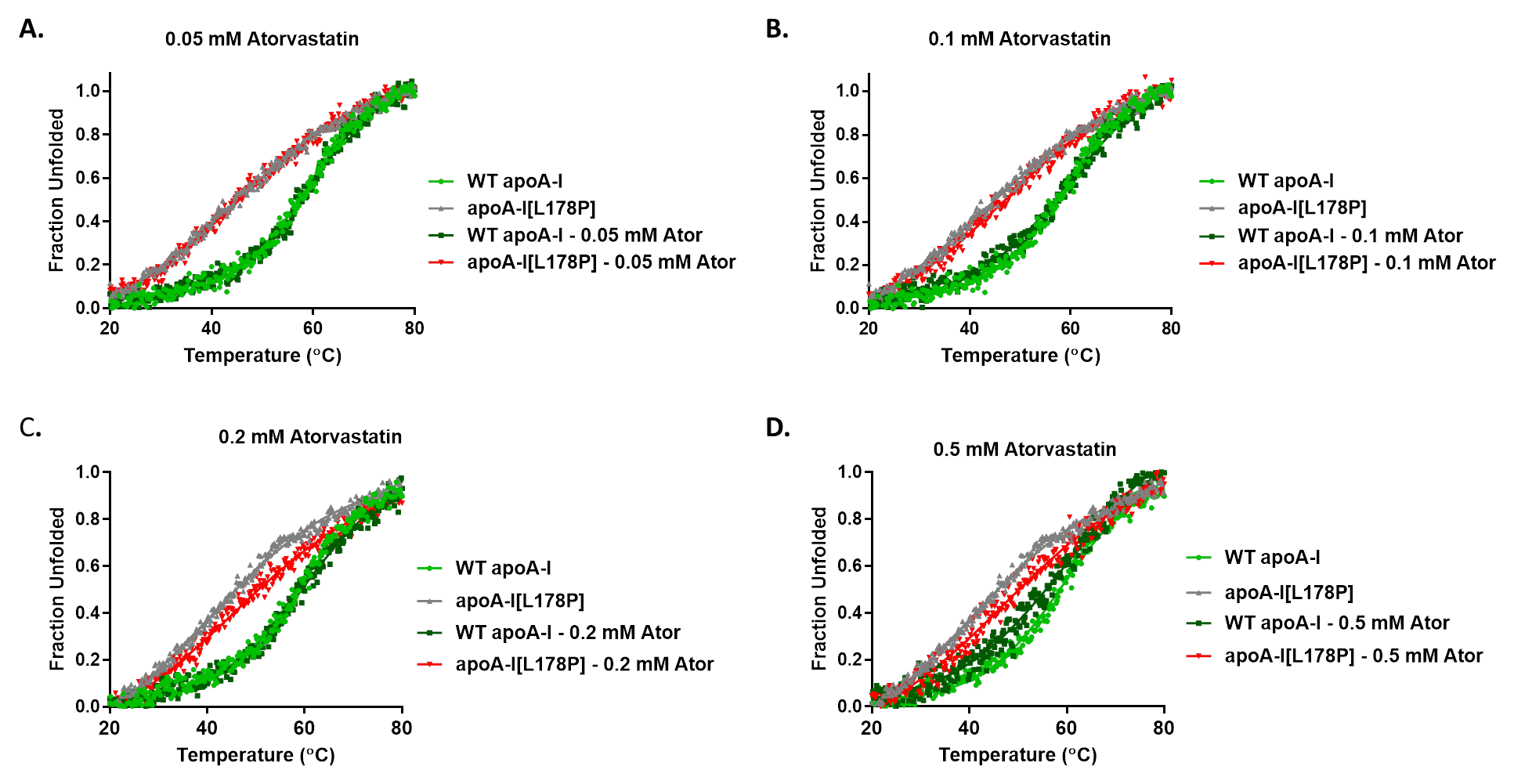


**Figure S8. Effect of Atorvastatin at increasing concentrations on thermal denaturation profiles WT apoA-I and apoA-I[L178P] (3.6 µM), monitored by CD spectroscopy.** Solid line indicates the fit of data to Boltzmann sigmoidal model. The *y* axis has been normalized to correspond to the fraction of the protein in the unfolded state.


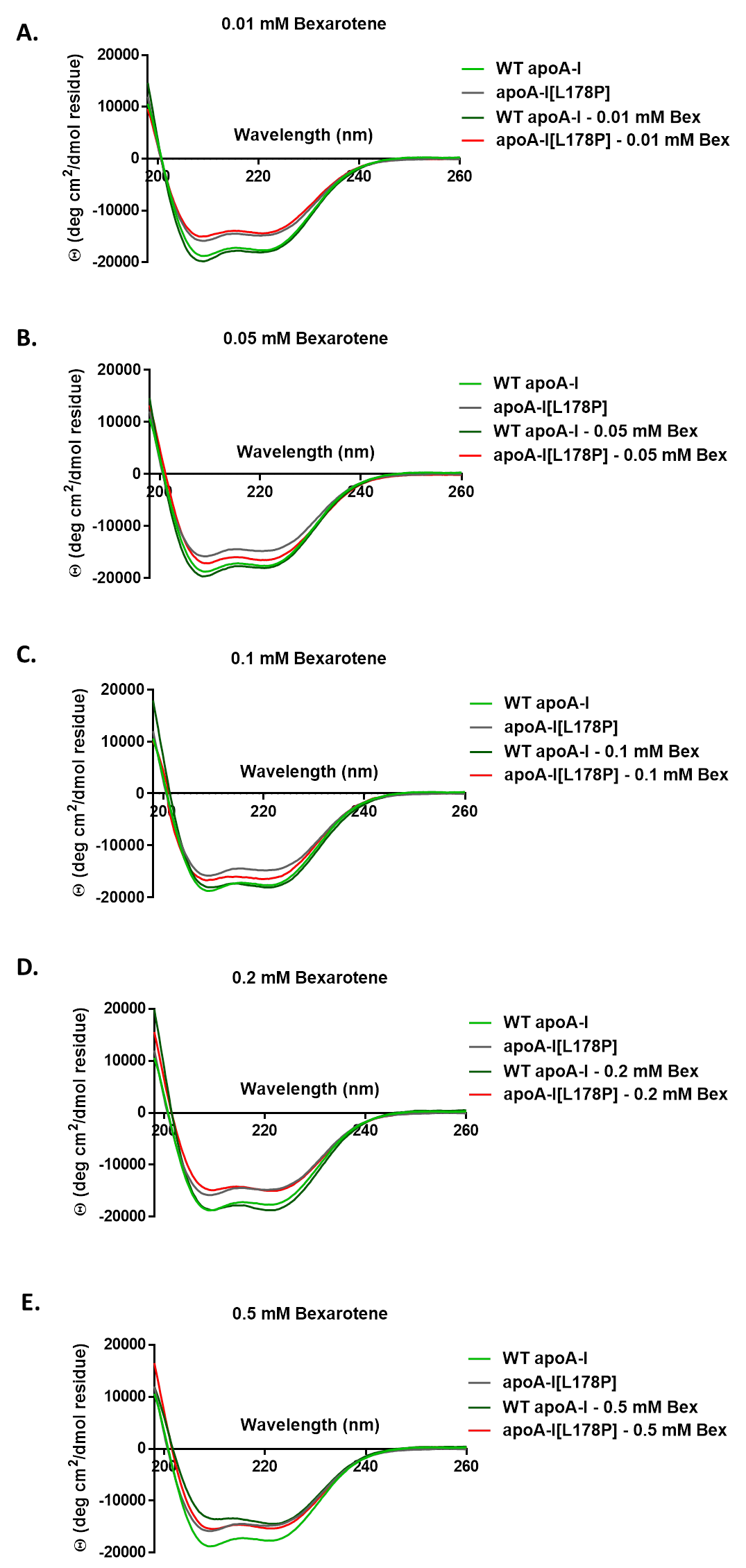


**Figure S9. Effect of Bexarotene, at increasing concentrations, on the far-UV CD spectra of WT apoA-I and apoA-I[L178P] (3.6 µM).**


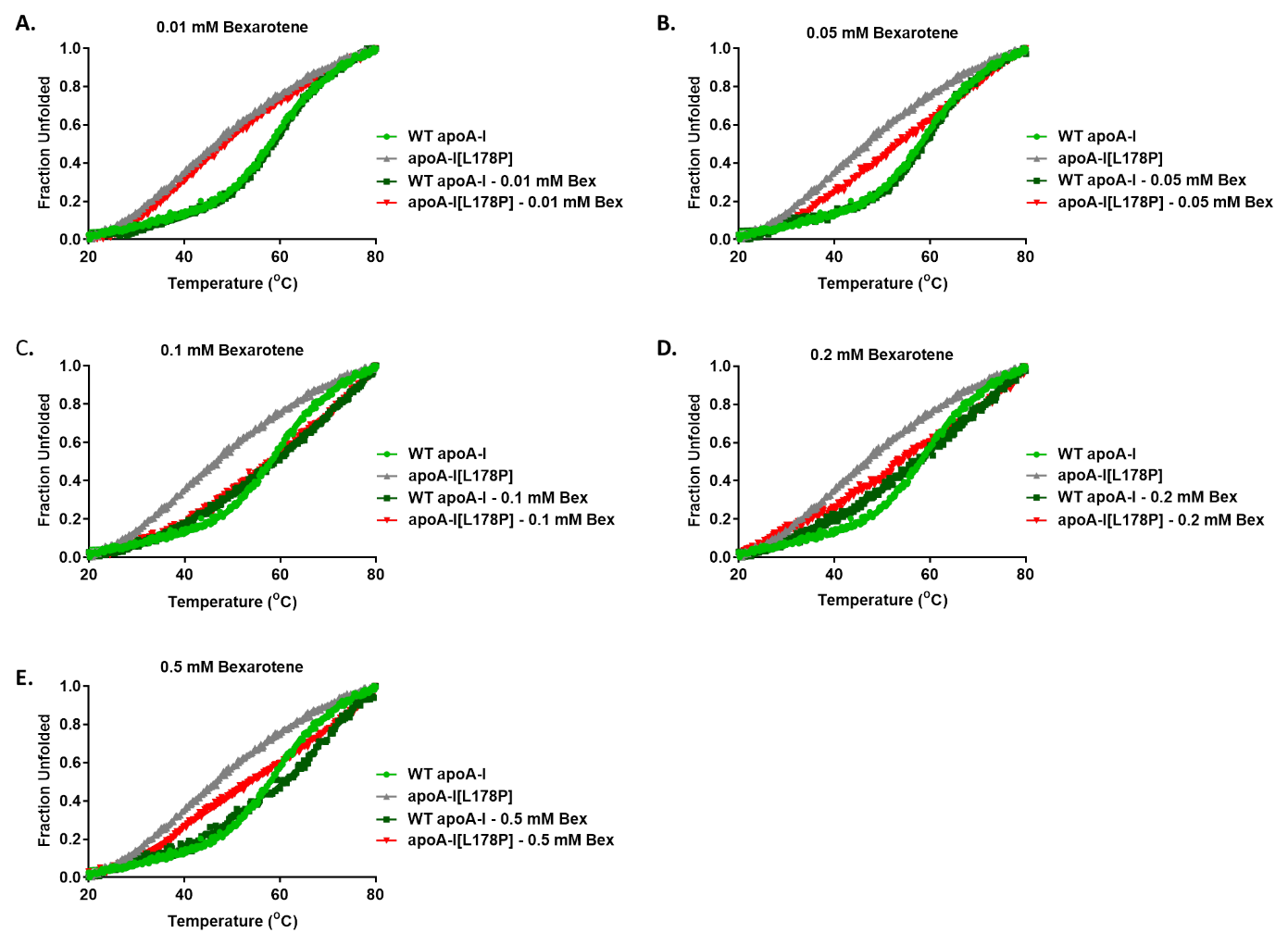


**Figure S10. Effect of Bexarotene at increasing concentrations on thermal denaturation of WT apoA-I and apoA-I[L178P] (3.6 µM), monitored by CD spectroscopy.** Solid line indicates the fit of data to Boltzmann sigmoidal model. The *y* axis has been normalized to correspond to the fraction of the protein in the unfolded state.


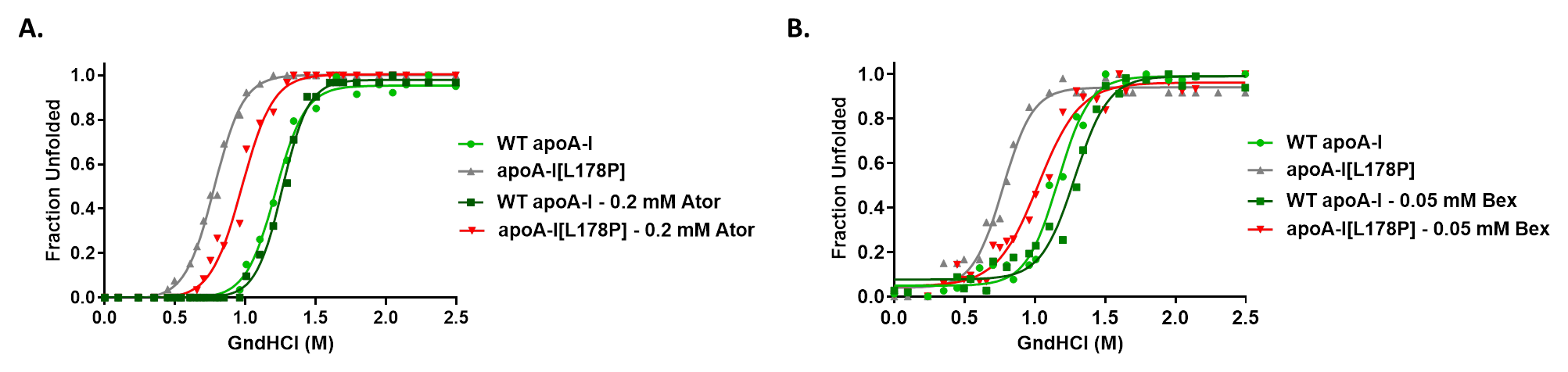


**Figure S11. Chemical denaturation profiles of WT apoA-I and apoA-I[L178P] (3.6 µM) in the absence or presence of 0.2 mM Atorvastatin (A) or 0.05 mM Bexarotene (B).** Solid line indicates the fit of data to Boltzmann sigmoidal model. The y axis has been normalized to correspond to the fraction of the protein in the unfolded state. Ator: Atorvastatin. Bex: Bexarotene.
